# Real‐Time Detection of Reduced Nitroreductase with a Reversible Fluorescent Probe

**DOI:** 10.1002/advs.202508689

**Published:** 2025-08-26

**Authors:** Sourav Sarkar, Anushree Shil, Yong Woong Jun, Yun Jae Yang, Ranran Cheng, Wenli Wu, Xuechen Li, Qiongzheng Hu, Kyo Han Ahn

**Affiliations:** ^1^ Department of Chemistry Pohang University of Science and Technology (POSTECH) Gyeongbuk 37673 Republic of Korea; ^2^ Department of Chemistry Korea Advanced Institute of Science and Technology (KAIST) Daejeon 34141 Republic of Korea; ^3^ Shandong Analysis and Test Center Qilu University of Technology (Shandong Academy of Sciences) Jinan 250014 China; ^4^ School of Chemistry and Chemical Engineering Qilu University of Technology (Shandong Academy of Sciences) Jinan 250353 China

**Keywords:** bio‐imaging, fluorescence probe, nitroreductase, reversible binding, senescence, tumor tissue

## Abstract

Nitroreductase (NTR), a class of flavin‐dependent redox enzymes, is a key biomarker for hypoxic tumors. Numerous fluorescent NTR probes have been developed to study hypoxia and associated tumors; however, they are reaction‐based and provide only static information on the accumulated enzyme activity at a given time. Reversible binding probes are needed to monitor the enzyme level in real time. Here, the first reversible binding probe is presented that selectively detects the active, reduced form of NTR (red‐NTR) with a fluorescence turn‐on response. This probe, a benzocoumarin dye functionalized with a (nitrobenzyl)pyridinium moiety, is stabilized through hydrogen bonding between its nitro group and the reduced cofactor flavin mononucleotide (FMNH_2_). This interaction suppresses both the enzymatic reduction and fluorescence quenching by photoinduced electron transfer. The probe selectively distinguishes red‐NTR from its oxidized form (ox‐NTR), allowing the observation of active enzyme levels in hypoxic cells, mouse tumor tissues, and cells undergoing premature senescence. The probe offers a unique and valuable tool for studying dynamic biological processes involving NTR under redox homeostasis.

## Graphical abstract

The first reversible binding probe (**rNTRp**) is presented that selectively detects the nitroreductase enzyme's active, reduced form (red‐NTR) with a fluorescence turn‐on response. Contrary to the conventional reaction‐based probes known to date, which provide only static information on the accumulated enzyme activity at a given time, **rNTRp** allows monitoring the active enzyme level in real time.

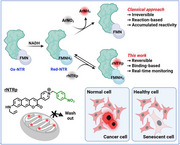

## 1 Introduction

**Figure 1:**
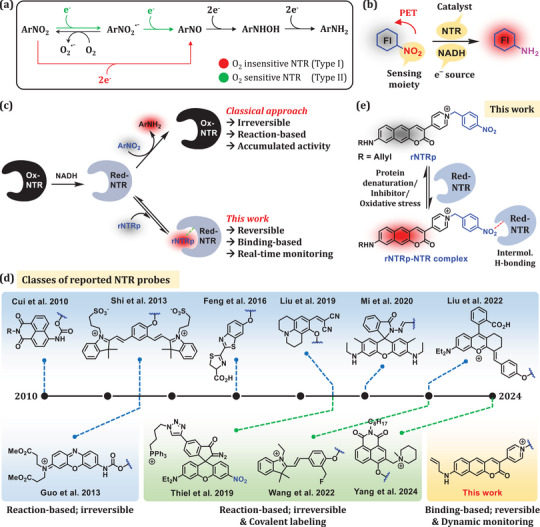
a) Reduction mechanisms of nitroaryl compounds by oxygen‐sensitive and ‐insensitive NTR. b) General design concept of reaction‐based probes for NTR. c) Sensing mechanisms of conventional reaction‐based probes and the first reversible binding probe, **rNTRp**. d) Classes of selected NTR probes. e) The sensing mechanism of **rNTRp**.

Nitroreductase (NTR), a class of flavin mononucleotide (FMN) cofactor‐dependent enzymes, catalyzes the reduction of nitroaromatic compounds to the corresponding amines aided with nicotinamide adenine dinucleotide (NADH).^[^ ^1^, ^2^, ^3^ ^]^ Each NADH molecule transfers two electrons to the FMN located in the binding pocket of NTR, generating FMNH_2_‐bearing NTR or reduced NTR (red‐NTR).^[^ ^2^ ^]^ Unlike the FMN bearing oxidized NTR (ox‐NTR), red‐NTR is active and converts nitroaromatic compounds into the corresponding hydroxylamines or further into the amines through a series of electron transfer steps (**Figure** **1**a). The reduction of nitroaromatic compounds into the corresponding hydroxylamines can proceed either through concerted (type I NTR) or stepwise (type II NTR) electron transfer mechanisms.^[^ ^3^ ^]^ The first electron transfer converts nitro compounds into the corresponding radical anion species, which can be rapidly oxidized back to the nitro compounds by molecular oxygen. Accordingly, type II NTR is oxygen‐sensitive and can reduce nitro compounds only under oxygen‐deficient/hypoxic conditions (hypoxia).^[^ ^4^, ^5^, ^6^ ^]^

The nitroreductase activity is elevated under hypoxia, a prominent feature of most solid tumors. Consequently, numerous fluorescent NTR probes have been developed (Figure 1b)^[^ ^4^, ^5^, ^6^, ^7^, ^8^, ^9^, ^10^, ^11^, ^12^, ^13^ ^]^ since the pioneering work by Cui et al in 2010.^[^ ^14^ ^]^ They are all reaction‐based/activatable probes based on nitroaryl compounds, which undergo the enzymatic reduction and eventually generate corresponding amines (Figure 1c). This chemical conversion causes a fluorescence signal change, mostly in the turn‐on mode. These reaction‐based probes, which can be readily designed by modifying the fluorophore, offer only static information on the accumulated enzyme activity at a given time.^[^ ^15^ ^]^ This innate property limits their use for dynamic monitoring of the enzyme levels. Additionally, the enzymatic product may diffuse to other spaces, which adds uncertainty in tracking down the enzyme in real time.^[^ ^16^ ^]^ Different from the reaction‐based probes, covalent labelling of NTR or nearby proteins with fluorophores triggered by the enzyme was used for the imaging of NTR activity in cells and tissues (Figure 1d).^[^ ^16^, ^17^, ^18^ ^]^ The labelling approach is also not suitable for the monitoring of intracellular NTR levels in real time. As red‐NTR and ox‐NTR are in an equilibrium inside the cell, monitoring the redox change at the active site is no longer possible once the labelling takes place. Therefore, for real‐time monitoring of NTR levels under dynamic redox homeostasis, there is an utmost demand for a fluorescent probe that detects the enzyme through a reversible binding process (Figure 1e). However, achieving a molecular probe specifically binding to the enzyme, along with a sufficient fluorescence signal change, remains a daunting task.

Here, we report a serendipitous discovery of the first reversible binding probe, which allows us to measure real‐time red‐NTR levels, rather than the accumulated activity levels detected by conventional reaction‐based probes. Notably, the probe, **rNTRp (r** eversible **NTR p** robe), selectively binds to red‐NTR rather than ox‐NTR, despite the structural similarity of their binding pockets.^[^ ^19^ ^]^

## 2 Results and Discussion

### 2.1 Probe Design

**Figure 2:**
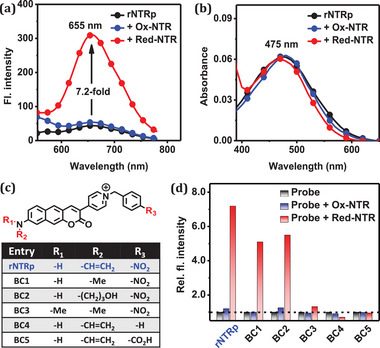
a,b) Emission and absorption spectra of **rNTRp** in the absence and presence of ox‐NTR (commercially available; in the oxidized form) and red‐NTR (ox‐NTR + NADH). (5.0 µm **rNTRp**, 4.0 µg mL^−1^ NTR, 100 µm NADH; pH 7.4 PBS). c) Structures of **rNTRp** homologs studied in this work. d) Relative fluorescence response of **rNTRp** and **BC1**−**5** to ox‐NTR and red‐NTR. (5.0 µm **rNTRp**, 4.0 µg mL^−1^ NTR, 100 µm NADH; pH 7.4 PBS). All data were recorded 60 min after incubation at 25 °C.

In the course of searching for an activatable ratiometric NTR probe based on benzo[*g*]coumarin dyes^[^ ^20^ ^]^ with deep tissue imaging capability under two‐photon microscopy,^[^ ^21^ ^]^ we synthesized **rNTRp** and evaluated its sensing properties toward NTR. We expected that **rNTRp** (λ_em_ = 655 nm, pH 7.4 PBS) would produce **PyBC1** (λ_em_ = 590 nm) after the enzymatic reduction of the nitro group followed by the fragmentation reaction (Scheme S1, Supporting Information).^[^ ^22^ ^]^ Contrary to our expectation, when **rNTRp** was treated with red‐NTR (a mixture of commercial ox‐NTR and NADH), no emission peak from the expected **PyBC1** (≈590 nm) was observed, although there was a turn‐on type fluorescence response with the maximum at 655 nm (**Figure** **2**a). The results suggested that the probe remained unreacted but bound to the enzyme. Furthermore, when **rNTRp** was treated with ox‐NTR in the absence of NADH, there was no signal change. There was no absorption spectral shift in comparison with the case of red‐NTR (Figure 2b). The results indicate that **rNTRp** recognizes only red‐NTR, but not ox‐NTR. The interaction between **rNTRp** and red‐NTR seems to be driven by non‐covalent binding.

To know the structural requirement for the interaction between **rNTRp** and red‐NTR, we investigated several other homologs of **rNTRp** with minimum structural perturbation (Scheme S2, Supporting Information). For this, we prepared **BC1‒5**, which had structural differences in both the electron‐donating amino group and the benzyl group connected to the pyridinium nucleus (Figure 2c). **BC1** and **BC2,** bearing methylamine and propanolamine donors, respectively, instead of the allylamine, also exhibited signal enhancement in the presence of red‐NTR (Figure 2d). However, **BC3** bearing a dimethylamine donor exhibited no considerable signal change. The results indicate that an amine donor capable of acting as a hydrogen bond donor is necessary for enzyme binding. Also, **BC4** and **BC5** bearing benzyl and (*p*‐carboxy)benzyl groups, respectively, instead of the (*p*‐nitro)benzyl group, did not produce any signal change. Thus, both the nitro and secondary amino groups seem to be necessary for the enzyme binding (See the molecular docking study below for further binding information).

### 2.2 Probing NTR in Solution

To optimize analysis conditions, at first, 5.0 µm **rNTRp** was subjected to 2.0 µg mL^−1^ NTR in the presence of varying concentrations of NADH (1.0−500 µM). At a fixed concentration of ox‐NTR (2.0 µg mL^−1^), **rNTRp** (5.0 µm) performed most efficiently with 100 µm NADH (Figure S1, Supporting Information).

**Figure 3:**
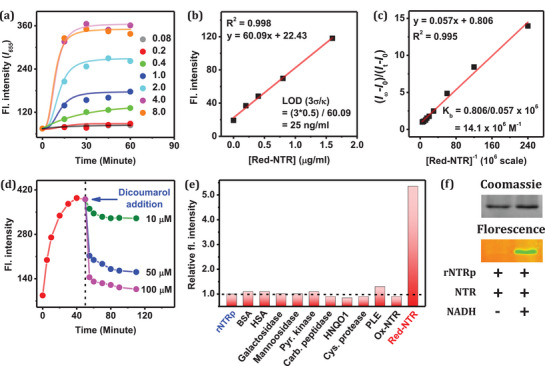
a) Fluorescence response of **rNTRp** (5.0 µm) to NTR (0.08–8.0 µg mL^−1^) in the presence of NADH (100 µm). b) Fluorescence intensity changes depending on red‐NTR (0–1.6 µg mL^−1^) at 5.0 µm **rNTRp** in pH 7.4 PBS, from which the limit of detection was calculated. c) Benesi–Hildebrand (B–H) plot, obtained from the titration of **rNTRp** (5.0 µm) with red‐NTR (0–0.21 µm) in pH 7.4 PBS at room temperature. d) Effect of dicoumarol (10–100 µm) for the binding of **rNTRp** (5.0 µm) to red‐NTR (4.0 µg mL^−1^). e) Fluorescence response of **rNTRp** (5.0 µm) to various proteins (BSA, HSA, galactosidase, mannosidase, pyruvate kinase, carboxypeptidase, NQO1, cysteine protease, PLE, ox‐NTR, and red‐NTR; each at 5.0 µg mL^−1^ in pH 7.4 PBS). The intensity data were recorded 60 min after mixing at 25 °C under excitation at 475 nm. f) SDS‐PAGE data, observed under UV excitation or by Coomassie staining. **rNTRp** (50 µm) and ox‐NTR (200 µg mL^−1^) were incubated for 1 h, in the absence and presence of NADH (1.0 mm), which was run in a 12% gel for 75 min (130 Volt).

Then, **rNTRp** was subjected to varying concentrations of NTR (0.08–8.0 µg mL^−1^) in the presence of 100 µm NADH, and the resulting fluorescence was monitored (**Figure** **3**a). The signal was saturated at 4.0 µg mL^−1^ NTR. The optimized conditions, 100 µm NADH, 4.0 µg mL^−1^ NTR, and 5.0 µm **rNTRp**, were thus used for the following assays. When 5.0 µm **rNTRp** was treated with 4.0 µg mL^−1^ red‐NTR, signal saturation was reached after 30 min (Figure S2, Supporting Information).

The fluorescence polarization experiment led to no change in the polarization value when the probe was treated with increasing concentrations of ox‐NTR. This result again supports that the probe does not interact with ox‐NTR (Figure S3, Supporting Information).

The calculated limit of detection (LOD) of **rNTRp** for red‐NTR was 25 ng mL^−1^, comparable to the values reported with other reaction‐based NTR probes (Figure 3b). The binding constant, determined in pH 7.4 buffer at 25 °C using the Benesi–Hildebrand equation, was 14.1 × 10⁶ m ^−1^, which indicates a strong affinity of **rNTRp** to the binding pocket of red‐NTR (Figure 3c). The binding of **rNTRp** to the active site of red‐NTR was confirmed through an inhibitor assay. Adding dicoumarol, a molecule that binds to the active site of NTR, caused a rapid decrease in the fluorescence intensity (Figure 3d). Note that dicoumarol is also known to inhibit the NADH‐dependent electron transfer pathway, which is essential for the conversion of inactive ox‐NTR into its active form, red‐NTR.^[^ ^23^ ^]^ To investigate the mechanism underlying dicoumarol‐induced fluorescence quenching of **rNTRp**, we designed two experiments by varying the order of addition of NADH, **rNTRp**, and dicoumarol to ox‐NTR:

i) ox‐NTR → NADH → **rNTRp** → dicoumarol

ii) ox‐NTR → dicoumarol → NADH → **rNTRp**

In sequence (i), dicoumarol is introduced last, allowing it to interact with the pre‐formed red‐NTR–**rNTRp** complex. Under these conditions, dicoumarol induced significant fluorescence quenching at higher concentrations (50–100 µm) (Figure 3d), but had minimal effect at lower concentrations (5–10 µm) (Figure S4a, Supporting Information). This suggests that dicoumarol can act as a competitor, displacing **rNTRp** from its binding site on red‐NTR at elevated concentrations. In contrast, in sequence (ii), dicoumarol is added before adding NADH and **rNTRp**, allowing it to interact with ox‐NTR. Here, even at low concentrations of dicoumarol, a substantial decrease in fluorescence resulted (Figure S4b, Supporting Information), which indicates that dicoumarol binds to ox‐NTR and prevents its reduction by NADH. These results support the dual role of dicoumarol: 1) it effectively inhibits the NADH‐mediated reduction of ox‐NTR to red‐NTR, and 2) at higher concentrations, it can compete with **rNTRp** for binding to red‐NTR, thereby displacing the probe and quenching fluorescence.

Next, the fluorescence response of **rNTRp** to various biologically relevant enzymes was examined. For this, 5.0 µm **rNTRp** was individually incubated with bovine serum albumin (BSA), human serum albumin (HSA), galactosidase, mannosidase, pyruvate kinase, carboxypeptidase, quinone oxidoreductase (HNQO1), cysteine protease, pig liver esterase (PLE), and ox‐NTR (5.0 µg mL^−1^ each) in pH 7.4 PBS (Figure 3e). No significant fluorescence signal change was observed in any of these cases. The exceptional selectivity of **rNTRp** for red‐NTR over ox‐NTR was further confirmed through SDS‐PAGE. In this experiment, **rNTRp** (50 µm) was incubated with either ox‐NTR or red‐NTR (200 µg mL^−1^) for 1 h before being subjected to SDS‐PAGE. A strong emission band was observed for red‐NTR while no emission was observed for ox‐NTR, which indicates that **rNTRp** can be used for the selective labelling of red‐NTR (Figure 3f).

### 2.3 Further Binding Studies

**Figure 4:**
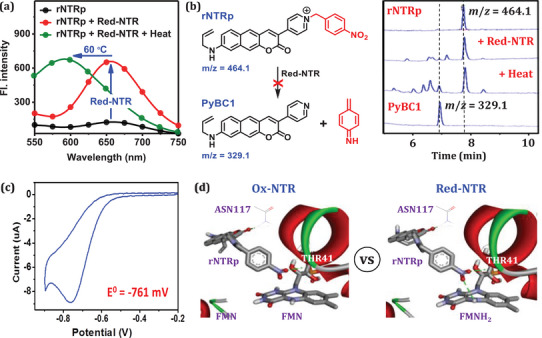
a) Emission spectral shift of the red‐NTR–rNTRp complex upon heating. b) LC‐MS analysis of the red–NTR–rNTRp complex upon heating. c) Cyclic voltammogram of rNTRp and its reduction potential. d) Molecular docking results of rNTRp with ox‐NTR (PDB code: 1KQC) and red‐NTR (PDB code: 1KQD), respectively.

Since heat can alter protein structures and affect the binding process, we examined the impact of temperature on the binding stability. A stable complex between red‐NTR and **rNTRp** was formed by incubating them at room temperature for 40 min, during which the fluorescence signal at 655 nm reached saturation. Upon heating the solution to 60 °C for 30 min, a distinct blue‐shifted emission peak emerged at 590 nm, replacing the original 655 nm peak (**Figure** **4**a). To investigate this phenomenon, we performed HPLC analysis for the probe and red‐NTR mixture both before and after heating. The results indicate that a small amount of the debenzylated product, **PyBC1**, is formed under these conditions (Figure 4b). This suggests that enzymatic reduction occurs during heating, although to a limited extent. The reduced product, **PyBC1**, is released from the enzyme and exhibits fluorescence at 590 nm. Notably, **PyBC1** emits significantly brighter fluorescence than the probe. The minor enzymatic reduction even at elevated temperature indicates that **rNTRp** is largely resistant to enzymatic reduction under typical biological conditions. This resistance is likely due to its significantly lower reduction potential (−761 mV, Figure 4c) compared to that of typical reaction‐based NTR probes, which generally exhibit potentials of −350 mV or higher and thus are readily reduced in cells.^[^ ^24^ ^]^

We conducted molecular docking studies to gain deeper insight into the molecular interactions between the probe and enzyme, using both oxidized (PDB code: 1KQC) and reduced (PDB code: 1KQD) nitroreductases, which contain FMN and FMNH_2_, respectively, in their active sites (Figure 4d). In the case of ox‐NTR (1KQC), the probe is positioned within the binding site, stabilized by two hydrogen bonds with the ASN117 and THR41 residues. In contrast, red‐NTR (1KQD) exhibits additional stabilization through an extra hydrogen bond with the central ring nitrogen of FMNH_2_. This difference may explain why **rNTRp** preferentially binds to the active site of red‐NTR, rather than that of ox‐NTR.

### 2.4 Reversibility

**Figure 5:**
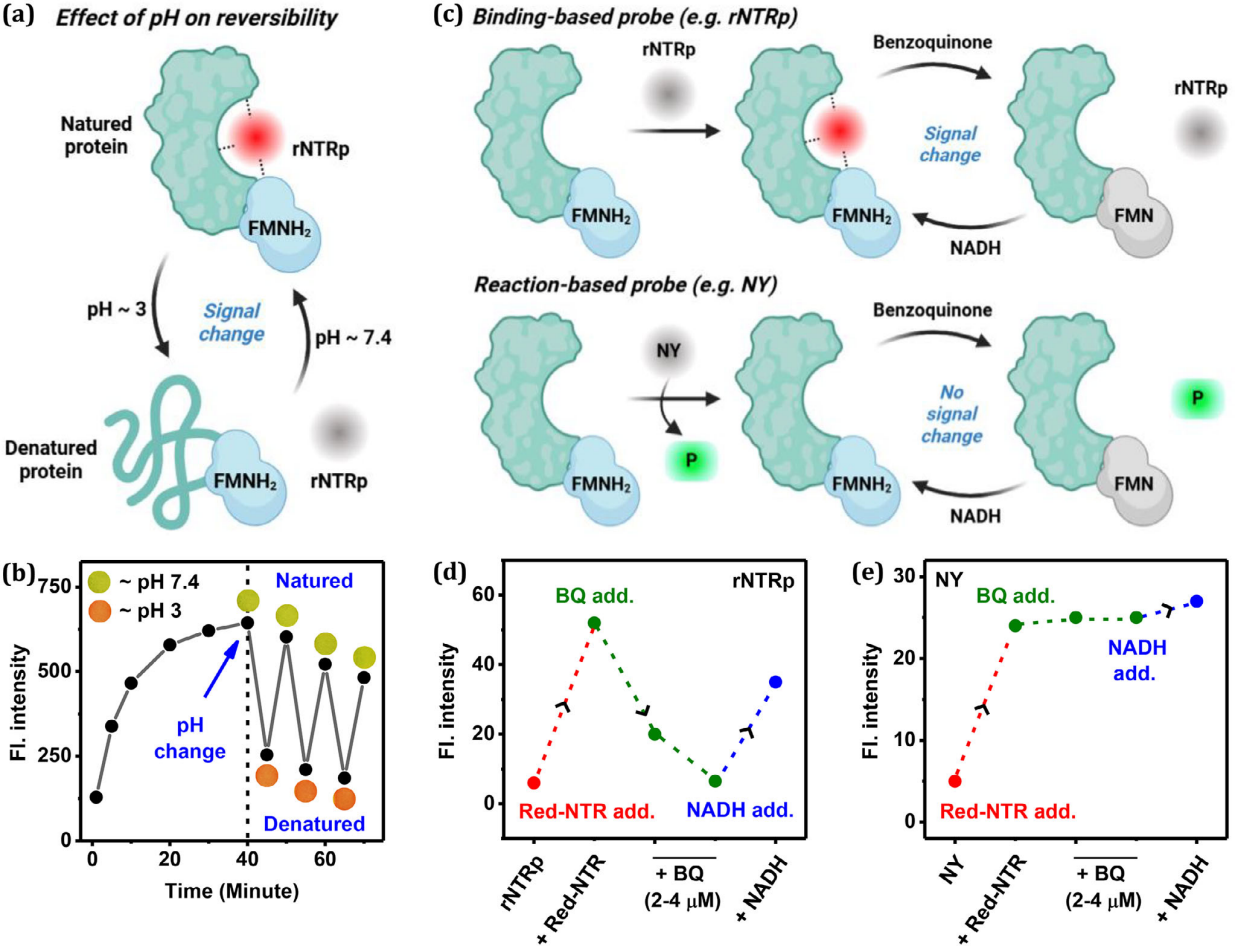
a) Anticipated pH effect on the red‐NTR− **rNTRp** complexation. b) Fluorescence intensity changes of a mixture of **rNTRp** (5.0 µm) and red‐NTR (4.0 µg mL^−1^), depending on the medium pH from 7.4 to ≈3 (measured using litmus paper; inset photos). c) Anticipated behavior of binding‐ and reaction‐based NTR probes in response to BQ and NADH. d,e) Effect of BQ and NADH on the sensing behaviour of NTR by **rNTRp**, in comparison with a reaction‐based probe **NY**: Each probe (5.0 µm) in pH 7.4 PBS was treated with red‐NTR (4.0 µg mL^−1^ ox‐NTR and 100 µm NADH) for 50 min, followed by BQ (2.0 and 4.0 µm) and NADH (100 µm) sequentially, and the resulting fluorescence intensity change was measured.

To further assess the reversibility of the probe‐enzyme binding, we varied the medium pH. At a neutral pH of ≈7.4, the **rNTRp**–red‐NTR complex exhibited strong fluorescence, whereas fluorescence intensity dropped significantly when the pH was lowered to 3.0 (**Figure** **5**a). Notably, the fluorescence on–off cycle could be repeated multiple times. Under acidic conditions, NTR undergoes denaturation, releasing the probe into the solution and causing the fluorescence to decrease. Upon restoring the pH to neutral, fluorescence was immediately recovered, indicative of reformation of the binding complex (Figure 5b). Please note that **rNTRp** itself in aqueous solution is not responsive to pH changes (Figure S5, Supporting Information). This reversible switching behavior further supports the dynamic and reversible binding nature of **rNTRp** to red‐NTR.

To demonstrate how a binding‐based probe like **rNTRp** enables real‐time monitoring of NTR—unlike conventional reaction‐based NTR probes—we investigated the effect of 1,4‐benzoquinone (BQ), an oxidizing agent that converts red‐NTR to ox‐NTR by oxidizing FMNH_2_. Both probe types were tested under these conditions (Figure 5c): As a reference, we used the well‐known reaction‐based NTR probe **NY**.^[^ ^16^ ^]^ Upon successive addition of BQ, a gradual decrease in fluorescence was observed with **rNTRp** (Figure 5d), consistent with its selective recognition of red‐NTR over ox‐NTR. This time‐dependent fluorescence quenching reflects real‐time changes in red‐NTR levels. Subsequent addition of NADH partially restored the fluorescence signal, as NADH reduces FMN in ox‐NTR to FMNH_2_, thereby regenerating active red‐NTR. In contrast, the fluorescence signal of **NY** remained largely unchanged after the addition of either BQ or NADH (Figure 5e), as reaction‐based probes, once enzymatically activated, are no longer capable of reflecting dynamic changes in NTR levels.

### 2.5 Study on the Sensing Mechanism

A major question was about the molecular interactions between **rNTRp** and red‐NTR that lead to fluorescence enhancement. We hypothesized that hydrogen bonding interactions could modulate frontier molecular orbital (FMO) energies, potentially inhibiting the photoinduced electron transfer (PET) quenching process associated with the probe's nitro group. Indeed, **rNTRp** forms hydrogen bonding not only with ASN119 and THR41 but also with FMNH_2_ inside the binding pocket of red‐NTR (Figure 4d). With the help of Gaussian calculations, we found that the formation of the H‐bonding between the nitro group and FMNH_2_ has a significant impact on the molecular orbital energies of **rNTRp**.

**Figure 6:**
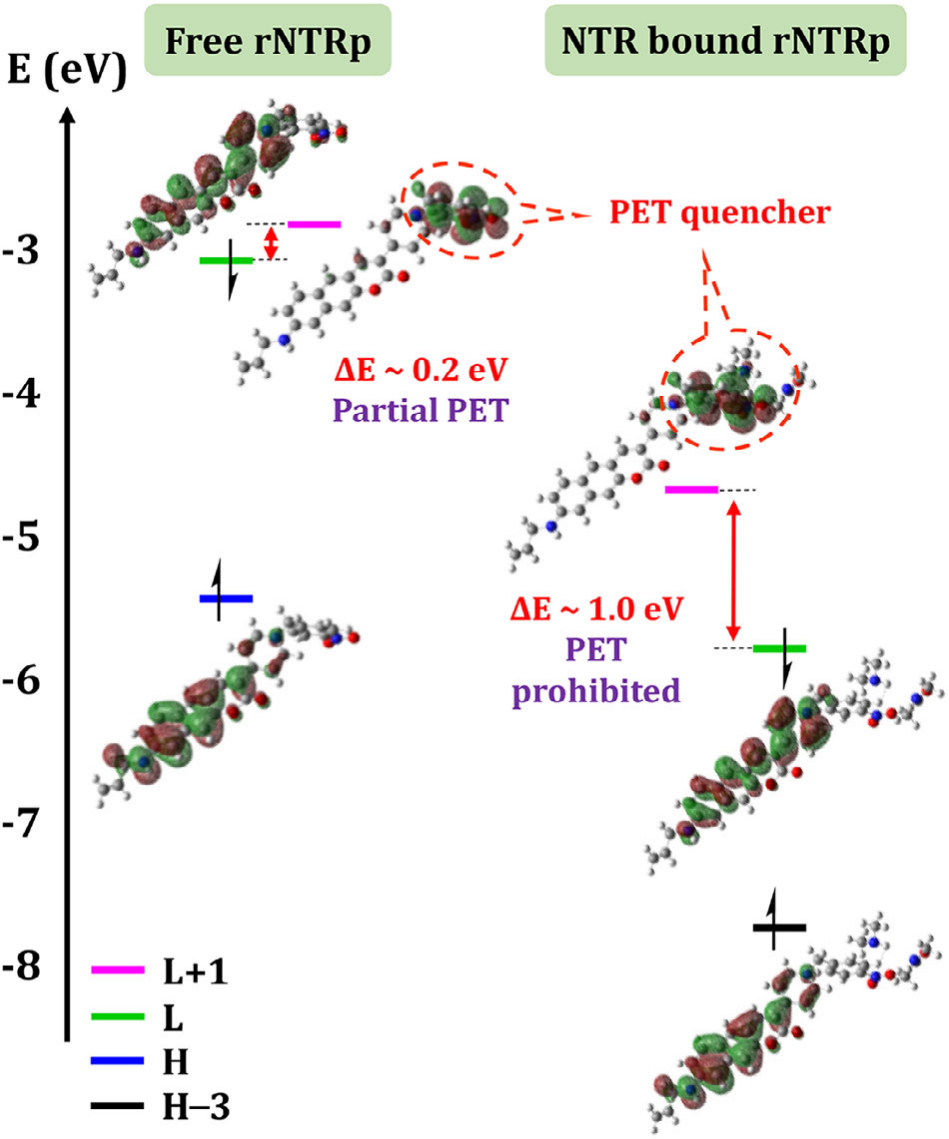
TD–DFT calculation data for **rNTRp** hydrogen‐bonded to red‐NTR (to the FMNH_2_ and THR41 residues; here, fragments of the residue were used to mimic the binding state), supporting the inhibition of PET quenching in the latter case; calculated using density functional B3PW91 and 6–31G(d,p) basis sets, water as medium.

**rNTRp** is essentially nonfluorescent in an aqueous solution, plausibly due to the partial oxidative PET quenching by the nitro group (**Figure** **6**). Contrarily, the H‐bonding between the nitro group and the FMNH_2_ of red‐NTR destabilizes the LUMO+1 energy level of the nitroaryl moiety, which hampers the oxidative PET process. Accordingly, **rNTRp** upon binding to red‐NTR emits fluorescence. In brief, we demonstrated the unique and promising detection capabilities of **rNTRp** both in solution and in silico. We subsequently evaluated its applicability for imaging in biological systems, as described below.

### 2.6 Intracellular Detection of Red‐NTR

As a donor‐acceptor type fluorophore, **rNTRp** exhibits negligible fluorescence in aqueous media (Φ_PBS_ = 0.008, Nile blue in ethanol as reference). In contrast, it emits strongly in non‐polar organic solvents (Φ_Dioxane_ = 0.35). Its emission spectra exhibited only a 25 nm red‐shift when the medium polarity changed from non‐polar dioxane to highly polar pH 7.4 PBS (Figure S6, Supporting Information). This polarity‐resistant property enabled us to predict the cellular emission maximum of **rNTRp** to be between 630 and 655 nm.^[^ ^25^, ^26^, ^27^, ^28^ ^]^ In fact, the observed emission maximum of **rNTRp** in A549 cells was 655 nm, which indicates no significant discrepancy in its optical properties between the solution and the cellular environment. The probe also exhibited high photostability even under continuous UV irradiation at 365 nm for 10 min (Figure S7, Supporting Information).

**Figure 7:**
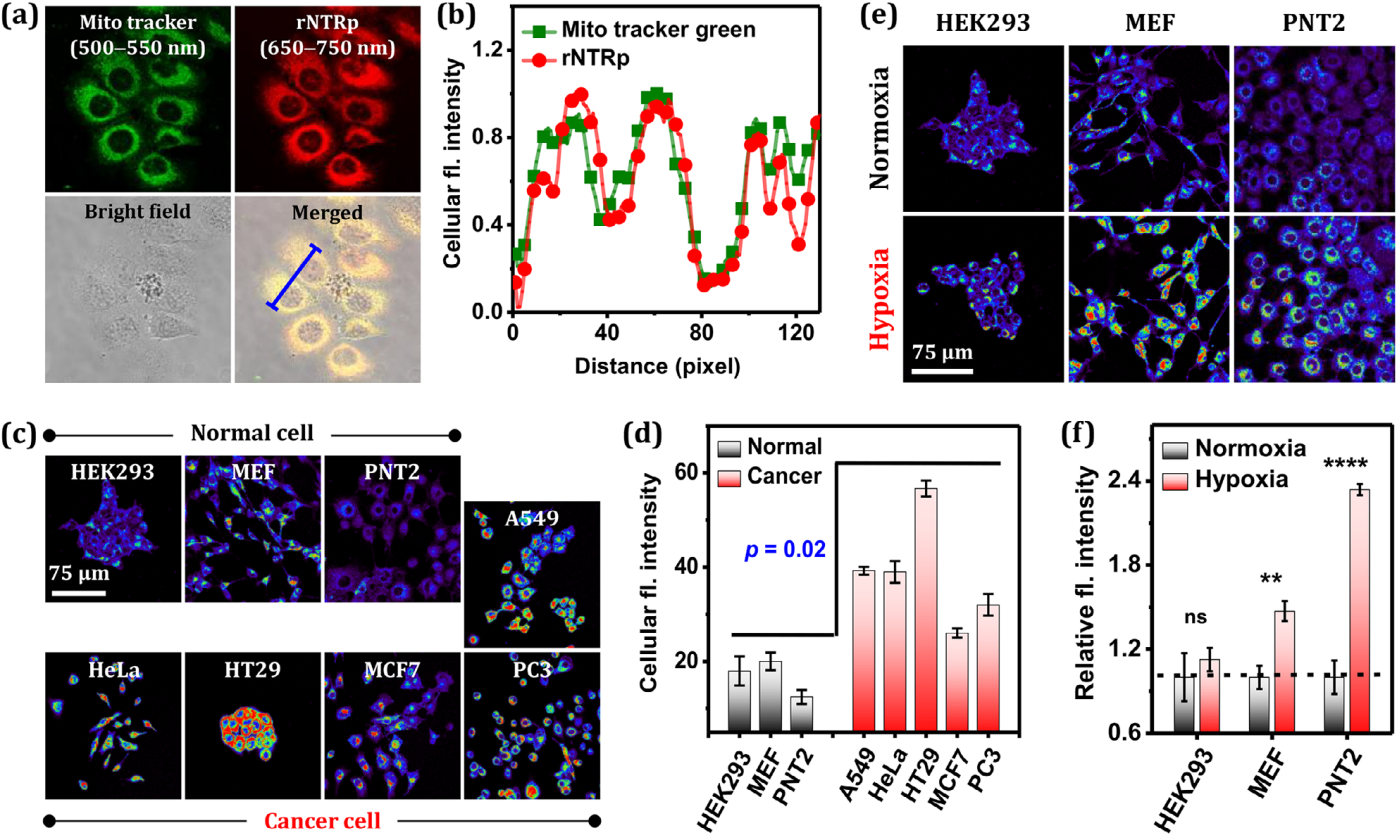
a) Colocalization data for rNTRp. A549 cells were co‐incubated with Mitogreen (mitochondria tracker, 500 nm) and rNTRp (5.0 µm) for 1 h and then imaged. CLSM images were obtained under excitation at 488 nm and emission collection through Mitogreen (500–550 nm, green) and rNTRp (650–750 nm, red) channels. b) Normalized fluorescence intensity plots against the ROI length (blue line in the merged image, Figure 7a). c) Images of three normal and five carcinoma cell lines incubated with rNTRp (5.0 µm) for 1 h, obtained under excitation at 488 nm and emission collection through 575–700 nm. d) Comparison of the cellular fluorescence intensities of (Figure 7c). e) Images of the normal cells under normoxic and hypoxic (created by the physical O_2_ depletion method for 1 h) conditions. f) Comparison of the cellular fluorescence intensities of (7e).

Next, we first examined the subcellular colocalization of **rNTRp**. Owing to its cationic and hydrophobic nature, **rNTRp** may localize in mitochondria.^[^ ^22^ ^]^ For this, A549 cells were co‐incubated with Mito‐green and **rNTRp** for 1 h and then subjected to fluorescence imaging. A Pearson's correlation coefficient (PCC) of 0.88 and a perfectly overlapped region‐of‐interest (ROI) intensity profile indicate that **rNTRp** localizes in mitochondria (**Figure** **7**a,b).

**rNTRp** produced bright fluorescence from mitochondria surrounding the nucleus (see the merged image) (Figure S8, Supporting Information). In contrast, **BC3**—a homolog of **rNTRp** containing the *N*,*N*‐dimethylamino donor—exhibited negligible fluorescence in the cellular environment, indicative of a lack of binding to red‐NTR.

Conventional NTR probes have been used to detect the conversion of nitro groups to corresponding amino groups under hypoxic conditions, reflecting the accumulated enzyme activity over time. However, real‐time monitoring of red‐NTR levels has not been achieved due to the absence of a suitable probe. First, we used **rNTRp** to compare real‐time cellular red‐NTR levels between cancer cells (A549, HeLa, HT29, MCF7, and PC3) and normal cells (HEK293, MEF, and PNT2) (Figure 7c,d). The cancer cells exhibited stronger fluorescence than the normal cells, indicative of elevated red‐NTR levels in the cancer cells. Of the cell lines screened, red‐NTR levels were lowest in PNT2 and highest in HT29. These results are consistent with previous reports,^[^ ^29^, ^30^ ^]^ which indicate that cancer cells possess relatively higher NTR levels and, consequently, greater enzymatic activity.

Next, we performed cellular imaging experiments to examine the impact of hypoxia on red‐NTR levels. Hypoxia is an oxygen‐deficient condition in cells, tissues, and organs. It is well established that cancer cells exhibit elevated NTR activity, particularly under hypoxic conditions.^[^ ^4^, ^5^, ^6^ ^]^ However, the effect of hypoxia on normal cells has been rarely explored using conventional NTR probes. To address this, we treated normal HEK293, MEF, and PNT2 cells with 5.0 µm **rNTRp** and subjected them to hypoxic conditions. A significant increase in fluorescence intensity was observed in PNT2 and MEF cells, whereas minimal or no change was observed in the case of HEK293 cells (Figure 7e,f). These results suggest that hypoxia induces higher red‐NTR levels in MEF and PNT2 cells. Luo et al. concluded that during short‐term hypoxia, the total intracellular NTR level remains unchanged.^[^ ^13^ ^]^ Based on this, we infer that hypoxia upregulates red‐NTR levels while downregulating ox‐NTR, thereby maintaining a constant overall NTR concentration. The hypoxia‐resistant behavior observed in HEK293 cells suggests either an absence or a significantly lower level of NTR in these cells. A similar conclusion was previously drawn from accumulated enzyme activity data over a defined period.^[^ ^29^, ^31^ ^]^

**Figure 8:**
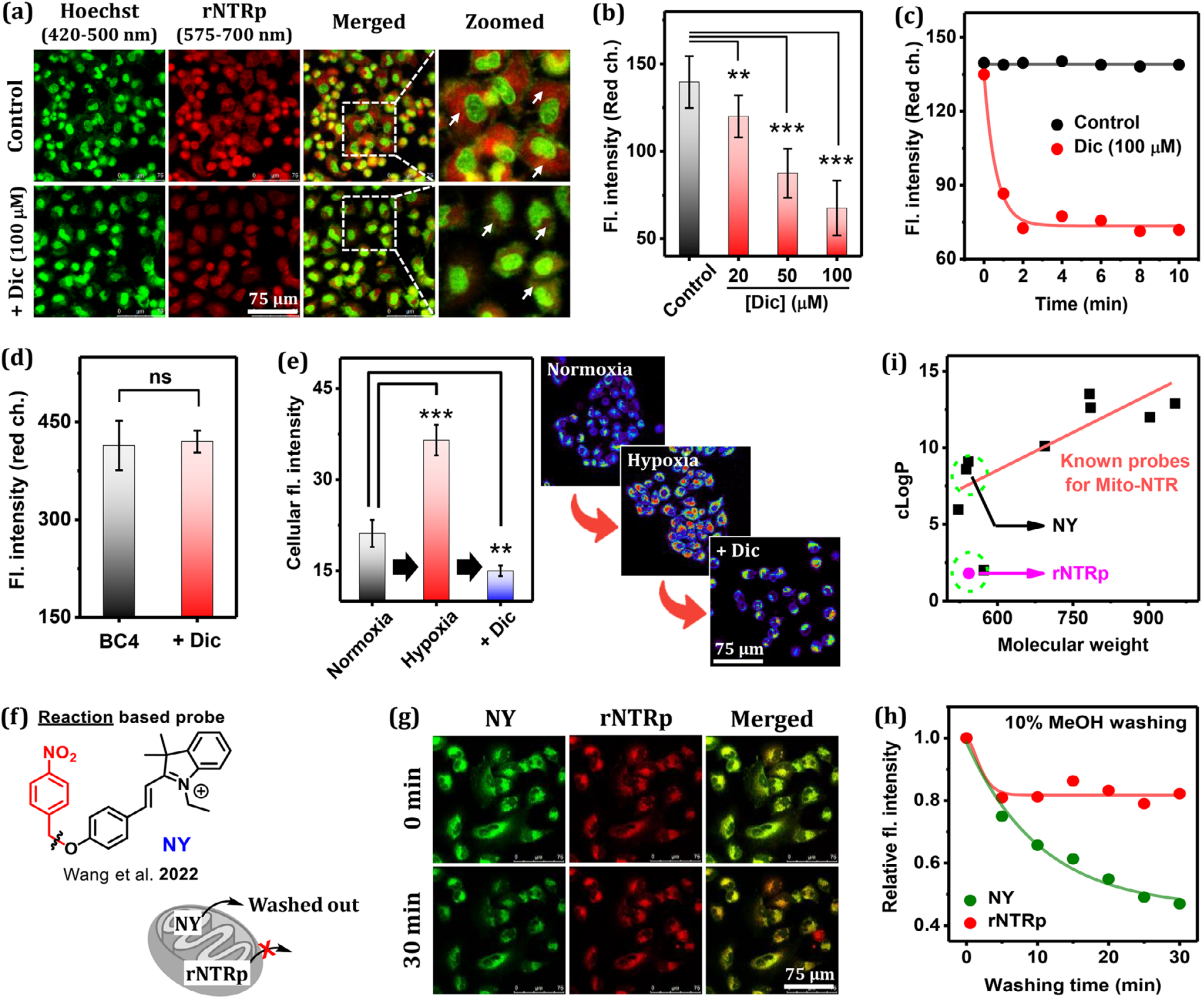
a) CLSM images of A549 cells, co‐incubated with **rNTRp** (5.0 µm) and Hoechst (10 µm) for 1 h under normoxic conditions or further incubated with dicoumarol (Dic, a nitroreductase inhibitor; incubation for 5 min), observed under excitation at 405 nm and emission collection through two channels: Hoechst (420–500 nm, green); **rNTRp** (575–700 nm, red). b) The red‐channel fluorescence intensity (representing red‐NTR) changes depending on the Dic concentration used. c) Time‐dependent emission intensity change of A549 cells incubated with **rNTRp** (5.0 µm), monitored after addition of 100 µm Dic. d) Fluorescence intensities of A549 cells incubated with **BC4** (5.0 µm, an **rNTRp** homolog missing the nitro group; 60 min‐incubation), in the absence and presence of Dic (100 µm), obtained from Figure S9 (Supporting Information) images. e) Fluorescence intensities of A549 cells incubated with **rNTRp** (5.0 µm) in normoxia, hypoxia (60 min under oxygen depletion), and Dic (100 µm, 10 min)‐treated cellular states. f) A representative reaction‐based, mitochondria‐targeting NTR probe, **NY**; the possibility of being washed out from the mitochondria. g) Images of A549 cells co‐incubated with **NY** (100 µm) and **rNTRp** (5.0 µm) for 6 h, observed before and 30 min after replacing the medium with pH 7.4 PBS containing 10% MeOH under 488 nm excitation. h) Relative fluorescence intensity changes after the medium replacement, monitored up to 30 min for the **NY**‐ and **rNTRp**‐incubated cells. i) Plot of cLogP versus molecular weights, for known mitochondria targeting NTR probes selected and **rNTRp**. ns (*p* > 0.1), ^*^(*p* < 0.1), ^**^(*p* < 0.01), ^***^(*p* < 0.001), ^****^(*p* < 0.0001). Error bars: mean ± SD (*n* = 3).

Next, we investigated the effect of an NTR inhibitor, dicoumarol (Dic), on the binding process. For this, A549 cells were co‐incubated with **rNTRp** (5.0 µm) and Hoechst (10 µm), a nucleus‐staining dye, and then emissions were collected through green and red channels, respectively (**Figure** **8**a). A bright red image surrounding nuclei was observed from mitochondrial red‐NTR.

When the cells were further treated with 100 µm dicoumarol, the red emission became dim. The results support that dicoumarol inhibits the probe binding (Figure 8b). Monitoring the time‐dependent emission intensity change upon the addition of dicoumarol led to fluorescence quenching within minutes (Figure 8c), which indicates a rapid displacement of the enzyme‐bound **rNTRp** by dicoumarol. We further investigated the inhibitory effect using **BC4**, a homolog of **rNTRp** that lacks the *p*‐nitro group and, consequently, the ability to bind. **BC4** was strongly emissive in cells, due to the absence of the nitro group. When the **BC4**‐incubated cells were further incubated with dicoumarol, the cellular fluorescence intensity remained unchanged, indicative of no inhibitory activity in this case (Figure 8d; Figure S8, Supporting Information). In other words, the fluorescence response of **rNTRp** to red‐NTR is due to its reversible binding to the active site.

Next, we compared the cellular red‐NTR levels depending on normoxic and hypoxic conditions using **rNTRp** (Figure 8e). We observed a ≈two‐fold higher level of red‐NTR in hypoxia than in normoxia. An added value of our probe over the conventional reaction‐based probes is its retaining property with the enzyme.^[^ ^32^ ^]^ To show this, we investigated **NY** as a control (Figure 8f).^[^ ^16^ ^]^ A549 cells were co‐incubated with **rNTRp** (5.0 µm) and **NY** (100 µm) for 6 h (such a long incubation time and high concentration were required to observe a detectable signal under normoxia in the case of the reaction‐based probe), and the medium was then replaced with pH 7.4 PBS containing 10% MeOH. The cellular emission intensities from **NY** and **rNTRp** were collected through 550–575 and 650–700 nm windows, respectively, to avoid signal cross‐talk (Figure S10, Supporting Information). The signal from **NY** gradually decreased, whereas that of **rNTRp** remained almost constant. Thus, the enzyme‐bound **rNTRp** is resistant to washout, unlike the known reaction‐based NTR probe (Figure 8g,h; Figure S11, Supporting Information). Interestingly, **rNTRp** exhibits a significantly lower cLogP (calculated lipophilicity) value of 1.8 compared to most mitochondria‐targeting NTR probes (cLogP = 9–13) (Figure 8i). For structurally related molecules, their molecular weights and cLogP values are proportionally related. High cLogP values can exhibit undesirable features, such as low water solubility, slow renal excretion, and high non‐specific binding to membranes and proteins. **rNTRp** thus offers advantages in that context.

### 2.7 Probing Red‐NTR in Tumor Tissue

**Figure 9:**
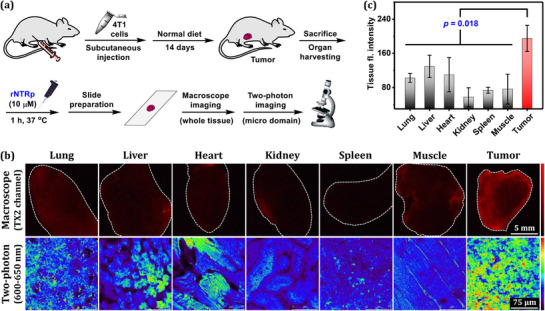
a) Schematic diagram for generating the allograft tumor model and experimental workflow for imaging. b) Macroscopic and two‐photon microscopic images of six mouse organs and the tumor site, incubated with **rNTRp** (5.0 µm, 30 min). Texas red filter was used for the macroscopic imaging: Exposure 1.9 s, gain 4.0, saturation 3.0, gamma 0.6, and zoom 6.9. Two‐photon images were obtained under excitation at 900 nm. Trans/gain: 40/40. c) Relative two‐photon tissue fluorescence intensity of the tumor and several organ tissues. Error bars: mean ± SD (*n* = 3).

The association between malignancy and elevated NTR activity has been well documented.^[^ ^4^ ^]^ We investigated whether **rNTRp** could differentiate tumor tissue from normal tissue. To this end, BALB/c mice were used to establish an allograft tumor model through subcutaneous injection of 4T1 cells (**Figure** **9**a). After 14 days, the mice were sacrificed, and tumor tissues along with various organs—including lung, liver, heart, kidney, spleen, and muscle—were harvested. The tissues were individually treated with **rNTRp** and subjected to macroscopic and two‐photon imaging. Macroscopic images revealed strong fluorescence in tumor tissue, while other organ tissues showed weak or negligible signals. This indicates that red‐NTR is upregulated in the tumor environment (Figure 9b).

Recently, benzocoumarin dyes have emerged as an outstanding platform for deep tissue imaging with minimal autofluorescence under two‐photon excitation.^[^ ^22^, ^26^, ^33^ ^]^ Leveraging these features, we captured images of A549 cells incubated with **rNTRp** under various two‐photon excitation conditions. We observed the highest cellular emission intensity at 900 nm two‐photon excitation (Figure S12, Supporting Information). A similar result was observed in **rNTRp**‐treated tissues from the tumor model mouse under two‐photon imaging, where the average emission intensity in tumor tissue was twice that of other organs (Figure 9c). This suggests that the level of red‐NTR in the tumor tissue is higher than that in other organ tissues.

### 2.8 Probing Red‐NTR in Senescent Cells

**Figure 10:**
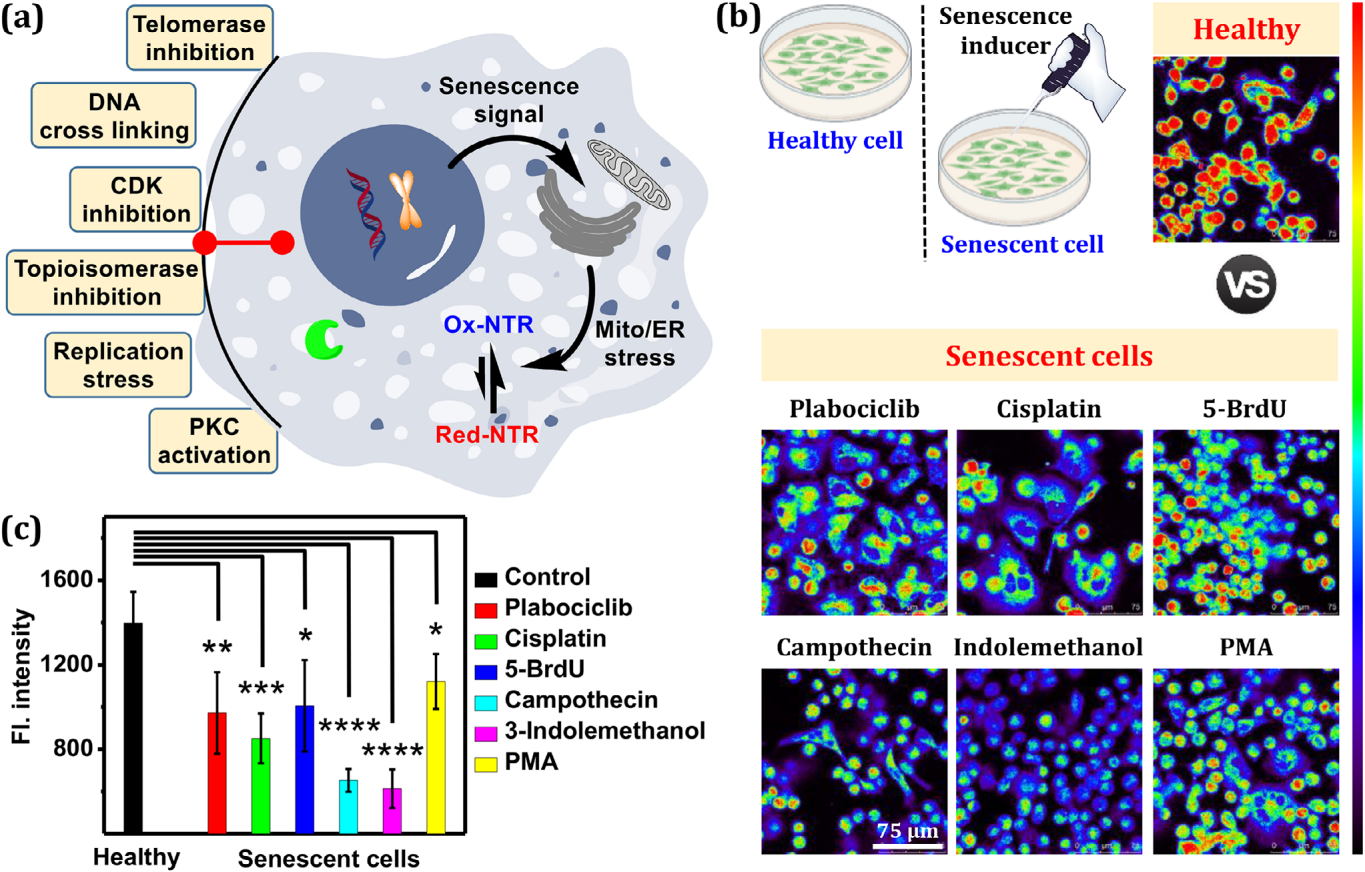
a) Different modes of senescence induction and plausible mechanisms of influencing ox‐NTR/red‐NTR equilibria. b) The procedure of chemical‐induced senescence induction and CLSM images of **rNTRp**‐treated healthy and senescent A549 cells. Palbociclib, 10 µm for 96 h; cisplatin, 15 µm for 72 h; 5‐BrdU, 100 µm for 48 h; camptothecin, 200 nm for 48 h; indole‐3‐methanol, 200 µm for 48 h; PMA, 1.0 µg mL^−1^ for 24 h. The cells were treated with 5.0 µm **rNTRp** for 1 h and then imaged with CLSM under excitation at 488 nm and emission collection through 575–700 nm. c) Relative cellular fluorescence intensity of healthy and senescent cells, measured from Figure 10b. ns (*p* > 0.1), ^*^(*p* < 0.1), ^**^(*p* < 0.01), ^***^(*p* < 0.001), and ^****^(*p* < 0.0001). Error bars: mean ± SD (*n* = 3).

Cellular senescence is an irreversible process, causing proliferative arrest.^[^ ^34^ ^]^ It is provoked when cells are exposed to a critical level of oncogenic stress. In this context, cellular senescence is probably an anticancer mechanism that prevents cells at risk from malignant transformation.^[^ ^35^ ^]^ It is known that senescence causes alterations in the proteome; however, how it affects the red‐NTR level has not been studied yet (**Figure** **10**a). Taking advantage of **rNTRp**’s unique sensing properties, we investigated the effect of chemically induced premature senescence on the red‐NTR level. Several chemicals that cause senescence in different modes were investigated, such as palbociclib (inhibits cyclin‐dependent kinase), cisplatin (cross‐links DNA), 5‐bromodeoxyuridine (5‐BrdU; induces replication stress), camptothecin (inhibits topoisomerase), indole‐3‐methanol (inhibits telomerase), and phorbol 12‐myristate 13‐acetate (PMA; activates protein kinase C).^[^ ^36^ ^]^ A549 cells were separately treated with these chemicals for the given period. The induction of senescence in the cells was confirmed by following the senescence‐associated β‐galactosidase (SA‐βgal), which is a widely accepted marker for cellular senescence, using the *O*‐nitrophenyl‐β‐D‐galactopyranoside (ONPG) assay (Figure S13, Supporting Information).^[^ ^37^ ^]^ Next, those cells were stained with **rNTRp,** and CLSM imaging was performed, which revealed strong fluorescence from healthy cells, but relatively weak fluorescence from the senescent cells (Figure 10b). In particular, the effect was prominent in both cases of camptothecin and indole‐3‐methanol (Figure 10c). The results suggest that the senescence influences the equilibrium between ox‐NTR and red‐NTR. Taken together, we can say that NTR activity in senescent cells is suppressed. The activity of many reductases, such as glutathione reductase, thioredoxin reductase, and ribonucleotide reductase activities, is also downregulated under senescence conditions, plausibly responding to the oxidative stress.^[^ ^38^, ^39^, ^40^ ^]^ To investigate whether the red‐NTR activity in senescent cells is associated with elevated ROS levels, we conducted a cellular ROS assay using DCFH‐DA (Figure S14a, Supporting Information).^[^ ^41^ ^]^ Cells were treated with various senescence inducers, including palbociclib, cisplatin, 5‐BrdU, camptothecin, indole‐3‐methanol, and PMA. Except for the cases of 5‐BrdU and camptothecin treatments, all senescent cells exhibited relatively elevated ROS levels (Figure S14b, Supporting Information). These results suggest a potential correlation between the red‐NTR level, as detected by **rNTRp**, and intracellular ROS activity measured by DCFH‐DA. However, ROS levels alone do not fully explain the observed changes in red‐NTR activity. Additional factors—such as upregulated NTR expression or stabilization of red‐NTR via alternative pathways—may also contribute. A detailed investigation of these mechanisms is beyond the scope of the present study.

## 3 Conclusion

We report the development of the first molecular probe, **rNTRp** that reversibly interacts with the active, reduced form of nitroreductases (red‐NTR). Unlike conventional probes that undergo irreversible enzymatic reduction, the probe binds reversibly to the substrate‐binding pocket through hydrogen bonding between its nitro group and FMNH_2_, the reduced cofactor of the enzyme. This interaction lowers the reduction potential of the nitro moiety, resisting the enzymatic reduction. The binding interactions also increase the LUMO+1 energy level, suppressing photo‐induced electron transfer quenching. Thus, the probe itself is weakly fluorescent but emits strong red fluorescence upon binding to red‐NTR in cells. Unlike reaction‐based probes that offer only static, accumulated data on enzyme activity, **rNTRp** enables real‐time monitoring of red‐NTR levels. Additionally, while reaction‐based probes generate reduction products that rapidly diffuse out of cells and compromise data reliability, enzyme‐bound **rNTRp** remains localized, ensuring more accurate measurements.

Importantly, **rNTRp** selectively distinguishes red‐NTR from its oxidized form (ox‐NTR), allowing us to measure active enzyme levels under various conditions. We demonstrated its applicability in detecting higher red‐NTR levels in cancer cell lines compared to normal cell lines, observing increased and variable red‐NTR levels in normal cells under hypoxic conditions versus normoxic conditions, observing elevated red‐NTR levels in tumor xenograft mouse tissues compared to other organ tissues, and identifying downregulated red‐NTR levels in cells undergoing premature senescence for the first time. Thus, **rNTRp** serves as a unique and valuable tool for studying nitroreductase‐associated biology under redox homeostasis conditions where the enzyme level can fluctuate in response to varying oxidative stress.

## 4 Experimental Section

### General Information

The chemical reagents were purchased from Aldrich or TCI. Commercially available reagents were used without further purification. The oxidized form of NTR was purchased from Sigma Aldrich (Product number: N9284). ^1^H and ^13^C NMR spectra were measured with a Bruker DPX‐500 spectrometer. Mass spectroscopic data were obtained from the Korea Basic Science Institute (Daegu) using a JEOL JMS high‐resolution mass spectrometer.

### Fluorescence Assays

UV–vis absorption spectra were obtained using an HP 8453 UV–Vis spectrophotometer. Fluorescence data were obtained with a Photon Technical International Fluorescence system using a 1.0 mL quartz cuvette. Stock solutions of each dye were prepared in DMSO (1.0 mm). MilliQ water was used to prepare all aqueous solutions. All measurements were performed at 25 °C.

### Cell Culture

Cells were obtained from the Korean Cell Line Bank and maintained in DMEM/RPMI supplemented with 10% (v/v) FBS and 1% (w/v) penicillin–streptomycin at 37 °C in a humidified atmosphere of 5% CO_2_ in air. Cells were passaged when they reached ≈80% confluency. Cells were incubated with **rNTRp** (5.0 µm) for a specific time before imaging. To generate the cellular hypoxic condition, dishes containing cells were filled with pH 7.4 PBS containing **rNTRp** and perfectly covered using parafilm. Sealed cell dishes were allowed to be incubated for a given time (1 or 2 h) and then fixed with formaldehyde.

### Bioimaging

One‐photon confocal and two‐photon fluorescent cellular images were recorded by Leica TCS SP5 II Advanced System equipped with a 25x objective lens (obj. HCX PL APO 25×/1.10 W CORR CS, Leica, Germany). Confocal imaging was obtained under 405 or 488 nm excitation, and emission was collected within the emission range of 575–700 nm. The two‐photon excitation wavelength was tuned to 900 nm, and emission light was collected within the emission range of 600–650 nm. Acquired images were processed using LAS AF Lite (Leica, Germany). Image intensity was calculated using ImagePro Plus.

### Docking Calculations

The binding affinity calculations between **rNTRp** and the oxidized and reduced forms of nitroreductase were carried out using software, AutoDock. The ligand (**rNTRp**) PDB file was generated from https://cactus.nci.nih.gov. The oxidized and two‐electron reduced nitroreductase structures were obtained from the protein data bank (https://www.rcsb.org) under code 1KQC and 1KQD, respectively. The docking results and figures were prepared using Discovery Studio.

### Preparation of Z‐Buffer

Standard Z‐buffer was prepared by dissolving 16.1 g of Na_2_HPO_4_ (120 mm), 5.5 g of NaH_2_PO_4_·H_2_O (80 mm), 0.75 g of KCl (10 mm), and 0.246 g of MgSO_4_·7H_2_O (1.0 mm) in 1 liter of distilled water. The pH was adjusted to 6.0 to selectively detect SA‐β‐gal. To trigger cell lysis, 0.1% triton‐X was added before use.

### ONPG Assay of Senescent Cells

The induction of cellular senescence was confirmed by detecting the presence of senescence‐associated β‐galactosidase activity, which is a widely accepted marker of cellular senescence. For that, we performed an ONPG (O‐nitrophenyl‐β‐D‐galactopyranoside) assay. In this assay, ONPG is cleaved by β‐galactosidase, producing a yellow compound, o‐nitrophenol, which can be measured spectrophotometrically at 420 nm. To perform the assay, A549 cells were first seeded on to a 96‐well plate and treated with senescence inducers, such as palbociclib (10 µm, for 96 h), cisplatin (15 µm, for 72 h), 5‐BrdU (100 µm, for 48 h), camptothecin (200 nm, for 48 h), indole‐3‐methanol (200 µm, for 48 h), and PMA (1.0 µg mL^−1^, for 24 h). After that, the cells were washed with PBS and then treated with pH 6.0 Z‐buffer containing ONPG (1.0 mg mL^−1^) for 3 h at 37 °C. Finally, the absorbance at 420 nm was measured and calibrated with cell viability to measure the level of β‐gal.

### DCFHDA Assay

Intracellular ROS levels were quantified using the fluorescent probe 2,7‐dichlorofluorescin diacetate (DCFH‐DA), which is a non‐fluorescent compound that produces strongly fluorescent DCF (λ_em_ = 530 nm) when oxidized by ROS inside the cell. This fluorescence observed is directly proportional to intracellular ROS levels. To perform the assay, A549 cells were first seeded onto a 96‐well plate and treated with senescence inducers: palbociclib, 10 µm for 96 h; cisplatin, 15 µm for 72 h; 5‐BrdU, 100 µm for 48 h; camptothecin, 200 nm for 48 h; indole‐3‐methanol, 200 µm for 48 h; PMA, 1.0 µg mL^−1^ for 24 h. After that, the cells were washed with PBS and treated with 10 µm DCFH‐DA in pH 7.4 PBS for 1 h at 37 °C. Fluorescence intensity was measured using a microplate reader (Ex/Em: 485/530 nm) and calibrated with the cell viability to measure the level of ROS.

### Statistical Analysis

The p values were calculated from the two‐tailed hypothesis using the T score and DF values for a pair of results. ns (*p* > 0.1), ^*^(*p* < 0.1), ^**^(*p* < 0.01), ^***^(*p* < 0.001), ^****^(*p* < 0.0001).

### Statement of Ethical Approval

The experimental procedures with mouse tissues were conducted according to the protocols approved by the Pohang University of Science and Technology Committee on Animal Research and by following the guidelines for using experimental animals by the Korean Academy of Medical Science (project No: POSTECH‐2023‐0074).

## Conflict of Interest

The authors declare no conflict of interest.

## Supporting information



Supporting Information

## Data Availability

The data that support the findings of this study are available in the supplementary material of this article.

## References

[1] D. W. Bryant , D. R. McCalla , M. Leeksma , P. Laneuville , Can. J. Microbiol. 1981, 27, 81. 7011517 10.1139/m81-013

[2] E. Johansson , G. N. Parkinson , W. A. Denny , S. Neidle , J. Med. Chem. 2003, 46, 4009. 12954054 10.1021/jm030843b

[3] F. J. Peterson , R. P. Mason , J. Hovsepian , J. L. Holtzman , J. Biol. Chem. 1979, 254, 4009. 374406

[4] B. Shang , Z. Yu , Z. Wang , Front. Pharmacol. 2024, 15, 1451517. 39101150 10.3389/fphar.2024.1451517 PMC11294179

[5] W. Qin , C. Xu , Y. Zhao , C. Yu , S. Shen , L. Li , W. Huang , Chin. Chem. Lett. 2018, 29, 1451.

[6] S. Das , H. K. Indurthi , P. Asati , D. K. Sharma , ChemistrySelect 2022, 7, 202102895.

[7] Y. Li , Y. Sun , J. Li , Q. Su , W. Yuan , Y. Dai , C. Han , Q. Wang , W. Feng , F. Li , J. Am. Chem. Soc. 2015, 137, 6407. 25923361 10.1021/jacs.5b04097

[8] D. Yang , H. Y. Tian , T. N. Zang , M. Li , Y. Zhou , J. F. Zhang , Sci. Rep. 2017, 7, 9174. 28835695 10.1038/s41598-017-09525-2 PMC5569069

[9] X. Meng , J. Zhang , Z. Sun , L. Zhou , G. Deng , S. Li , W. Li , P. Gong , L. Cai , Theranostics 2018, 8, 6025. 30613279 10.7150/thno.26607 PMC6299436

[10] K. H. Gebremedhin , Y. Li , Q. Yao , M. Xiao , F. Gao , J. Fan , J. Du , S. Long , X. Peng , J. Mater. Chem. B 2019, 7, 408. 32254728 10.1039/c8tb02635a

[11] C. Yu , S. Wang , C. Xu , Y. Ding , G. Zhang , N. Yang , Q. Wu , Q. Xiao , L. Wang , B. Fang , C. Pu , J. Ge , L. Gao , L. Li , S. Q. Yao , Adv. Healthcare Mater. 2022, 11, 2200400. 10.1002/adhm.202200400 35485404

[12] S. Zhang , M. Ma , C. Zhao , J. Li , L. Xu , Z. Zhang , Q. Diao , P. Ma , D. Song , Biosens. Bioelectron. 2024, 261, 116514. 38908291 10.1016/j.bios.2024.116514

[13] S. Luo , R. Zou , J. Wu , M. P. Landry , ACS Sens. 2017, 2, 1139. 28741347 10.1021/acssensors.7b00171 PMC10494911

[14] L. Cui , Y. Zhong , W. Zhu , Y. Xu , Q. Du , X. Wang , X. Qian , Y. Xiao , Org. Lett. 2011, 13, 928. 21268631 10.1021/ol102975t

[15] J. Zhan , W. Song , E. Ge , L. Dai , W. Lin , Coord. Chem. Rev. 2023, 493, 215321.

[16] S. Wang , W. Tan , W. Lang , H. Qian , S. Guo , L. Zhu , J. Ge , Anal. Chem. 2022, 94, 7272. 35549110 10.1021/acs.analchem.2c00512

[17] Z. Thiel , P. Rivera‐Fuentes , Angew. Chem., Int. Ed. 2019, 58, 11474. 10.1002/anie.201904700 31144369

[18] Y. J. Yang , Y. L. Jung , A. Shil , S. Sarkar , K. H. Ahn , Anal. Chem. 2024, 96, 11318. 38940602 10.1021/acs.analchem.4c01274

[19] C. A. Haynes , R. L. Koder , A. F. Miller , D. W. Rodgers , J. Biol. Chem. 2002, 277, 11513. 11805110 10.1074/jbc.M111334200

[20] M. Tasior , D. Kim , S. Singha , M. Krzeszewski , K. H. Ahn , D. T. Gryko , J. Mater. Chem. C 2015, 3, 1421.

[21] Y. W. Jun , H. R. Kim , Y. J. Reo , M. Dai , K. H. Ahn , Chem. Sci. 2017, 8, 7696. 29568432 10.1039/c7sc03362a PMC5851340

[22] H. R. Kim , S. Sarkar , K. H. Ahn , Chem. Asian J. 2022, 17, 202101317. 10.1002/asia.202101317 34962711

[23] R. L. Koder , A. F. Miller , BBA Protein Struct. Mol. Enzymol. 1998, 1387, 395.

[24] A. F. Miller , J. T. Park , K. L. Ferguson , W. Pitsawong , A. S. Bommarius , Molecules 2018, 23, 211. 29364838 10.3390/molecules23020211 PMC6017928

[25] S. Sarkar , A. Shil , Y. W. Jun , Y. J. Yang , W. Choi , S. Singha , K. H. Ahn , Adv. Funct. Mater. 2023, 33, 2304507.

[26] S. Sarkar , M. Santra , S. Singha , Y. W. Jun , Y. J. Reo , H. R. Kim , K. H. Ahn , J. Mater. Chem. B 2018, 6, 4446. 32254662 10.1039/c8tb01144c

[27] Y. J. Reo , Y. W. Jun , S. W. Cho , J. Jeon , H. Roh , S. Singha , M. Dai , S. Sarkar , H. R. Kim , S. Kim , Y. Jin , Y. L. Jung , Y. J. Yang , C. Ban , J. Joo , K. H. Ahn , Chem. Commun. 2020, 56, 10556. 10.1039/d0cc03586f 32785337

[28] S. Sarkar , A. Shil , S. Maity , Y. L. Jung , M. Dai , A. Acharya , K. H. Ahn , Angew. Chem., Int. Ed. 2023, 62, 202311168. 10.1002/anie.202311168 37700529

[29] Z. Li , X. Li , X. Gao , Y. Zhang , W. Shi , H. Ma , Anal. Chem. 2013, 85, 3926. 23506563 10.1021/ac400750r

[30] Z. Zhang , T. Lv , B. Tao , Z. Wen , Y. Xu , H. Li , F. Liu , S. Sun , Bioorg. Med. Chem. 2020, 28, 115280. 31899090 10.1016/j.bmc.2019.115280

[31] A. V. Sharrock , S. P. McManaway , M. H. Rich , J. S. Mumm , I. F. Hermans , M. Tercel , F. B. Pruijn , D. F. Ackerley , Front. Pharmacol. 2021, 12, 701456. 34163368 10.3389/fphar.2021.701456 PMC8215503

[32] Y. Hori , K. Kikuchi , Curr. Opin. Chem. Biol. 2013, 17, 644. 23743124 10.1016/j.cbpa.2013.05.015

[33] S. Sarkar , A. Shil , M. Nandy , S. Singha , Y. J. Reo , Y. J. Yang , K. H. Ahn , Anal. Chem. 2022, 94, 1373. 34990113 10.1021/acs.analchem.1c04646

[34] J. M. V. Deursen , Nature 2014, 509, 439.24848057

[35] J. Campisi , Annu. Rev. Physiol. 2012, 75, 685. 23140366 10.1146/annurev-physiol-030212-183653 PMC4166529

[36] N. V. Petrova , A. K. Velichko , S. V. Razin , O. L. Kantidze , Aging Cell 2016, 15, 999. 27628712 10.1111/acel.12518 PMC6398529

[37] F. Debacq‐Chainiaux , J. D. Erusalimsky , J. Campisi , O. Toussaint , Nat. Protoc. 2009, 4, 1798. 20010931 10.1038/nprot.2009.191

[38] G. A. Hazelton , C. A. Lang , Mech. Ageing. Dev. 1985, 29, 71. 3982084 10.1016/0047-6374(85)90048-x

[39] J. E. Dick , J. A. Wright , Mech. Ageing Dev. 1982, 20, 103. 6757589 10.1016/0047-6374(82)90062-8

[40] Y. H. Yoo , D. W. Kim , B. H. Chen , H. Sim , B. Kim , J. C. Lee , J. H. Ahn , Y. Park , J. H. Cho , I. J. Kang , M. H. Won , T. K. Lee , Lab. Anim. Res. 2021, 37, 11. 33676586 10.1186/s42826-021-00088-y PMC7937215

[41] D. Figueroa , M. Asaduzzaman , F. Young , J. Pharmacol. Toxicol. Methods. 2018, 94, 26. 29630935 10.1016/j.vascn.2018.03.007

