# Supplementary material for: Real‐Time Detection of Reduced Nitroreductase with a Reversible Fluorescent Probe
Source: Adv Sci (Weinh). 2025 Aug 26;12(42):e08689. doi: 10.1002/advs.202508689 (PMC12622434; doi:10.1002/advs.202508689)
Supplement: Supplementary file 1 — Supporting Information [file ADVS-12-e08689-s001.pdf]

Supporting Information

Real-Time Detection of Reduced Nitroreductase with a Reversible Fluorescent Probe

Sourav Sarkar, Anushree Shil, Yong Woong Jun, Yun Jae Yang, Ranran Cheng, Wenli Wu, Xuechen Li, Qiongzheng Hu,* and Kyo Han Ahn**

sourav@postech.ac.kr; huqz@qlu.edu.cn; ahn@postech.ac.kr

Supporting figures .... Page SI-2
Synthetic details .... Page SI-8
NMR characterization .... Page SI-9
HR-MS characterization .... Page SI-11
LC-MS characterization .... Page SI-12

1. Supporting figures

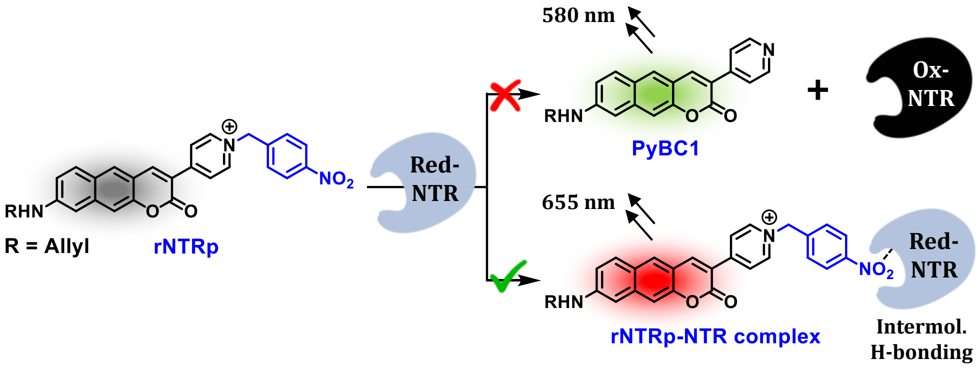

Scheme S1. Expected vs. observed response of **rNTRp** (5.0 μM) toward red-NTR (4.0 μg/mL NTR, 100 μM NADH; pH 7.4 PBS).

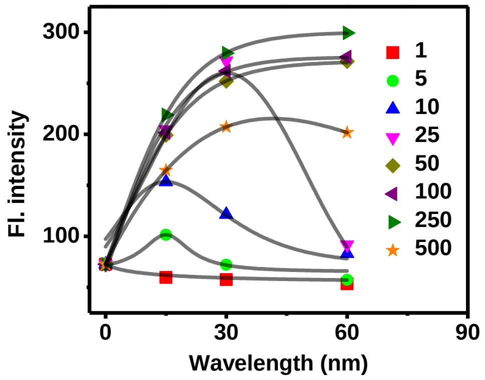

Figure S1. Response of **rNTRp** (5.0 μM) towards varying concentrations of NADH in the presence of NTR (2.0 μg/mL).

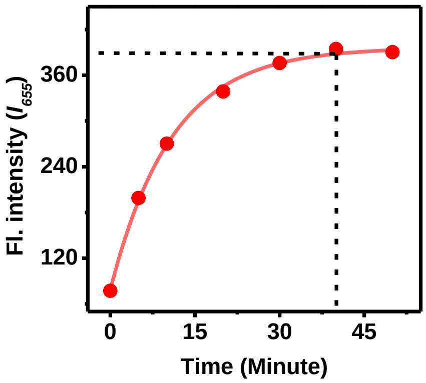

Figure S2. Time-dependent fluorescence intensity change, followed at the emission maxima. Data were recorded at room temperature using 5.0 μM **rNTRp**, 100 μM NADH, and 4.0 μg/mL NTR in pH 7.4 PBS.

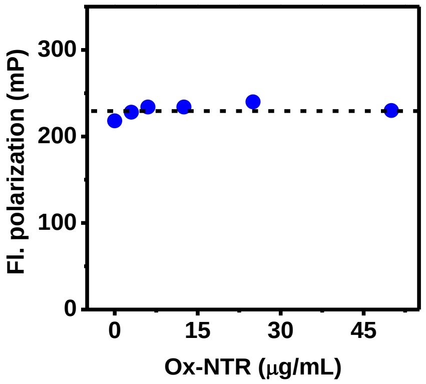

Figure S3. Fluorescence polarization change of **rNTRp** (5.0 μM) depending on concentrations of ox–NTR. The negligible change indicates little interaction between **rNTRp** and ox-NTR.

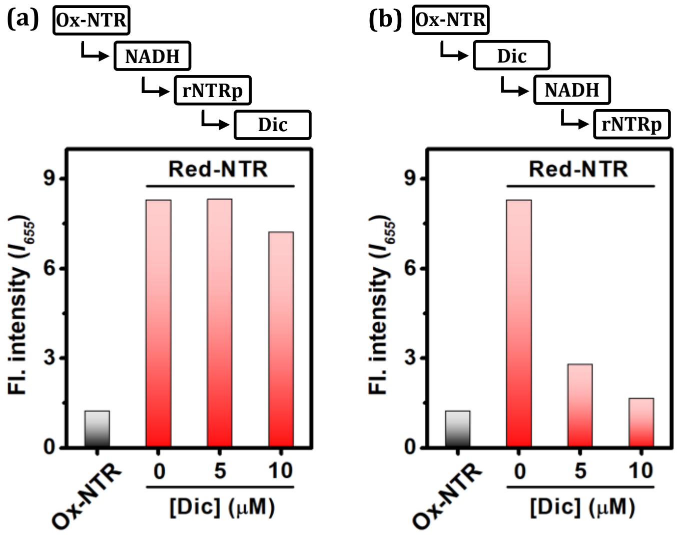

Figure S4. (a, b) Effect of the addition sequence of NADH, **rNTRp**, and dicoumarol on the fluorescence intensity. Ox-NTR, 4.0 μg/mL; NADH, 100 μM; Dicoumarol, 0–10 μM; **rNTRp**, 5.0 μM.

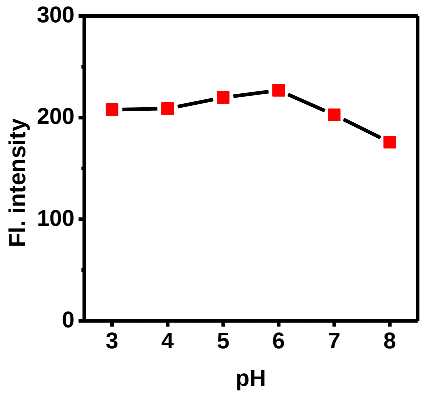

Figure S5. Effect of pH on the emission property of **rNTRp** (5.0 μM) in different universal buffer solutions. Intensity were obtained after 30 min incubation under 475 nm excitation.

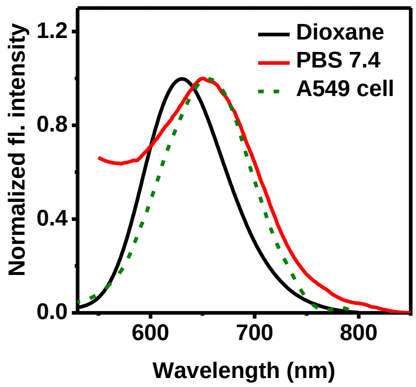

Figure S6. Comparison of emission spectra of **rNTRp** (5.0 μM), measured in dioxane (ε = 2.25 D), cell, and pH 7.4 PBS (ε = 80.5 D). Fluorescence spectra were obtained under excitation at their respective absorption maxima. The cellular spectrum was taken under excitation at 488 nm.

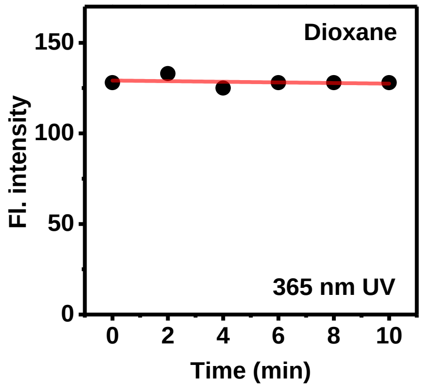

Figure S7. Photostability data of **rNTRp** (5.0 μM) in dioxane, measured under continuous UV irradiation at 365 nm.

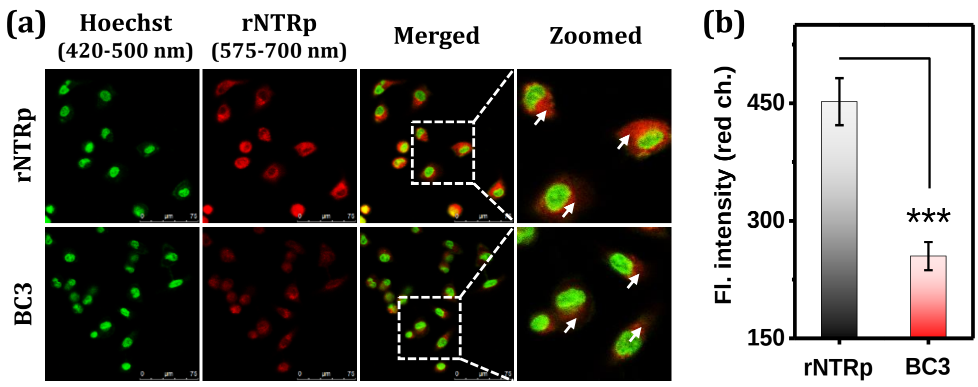

Figure S8. (a) CLSM images of A549 cells separately incubated with **rNTRp** and **BC3** (5.0 μM each) in the presence of Hoechst (10 μM) in normoxic conditions for 1 h. Images were obtained under excitation at 405 nm and emission collection in two channels: Hoechst in green (420–500 nm), both **rNTRp** and **BC3** in red (575–700 nm). (b) Comparison of the cellular emission intensities of **rNTRp** and **BC3**, observed through the red channel. ns ($p > 0.1$), *($p < 0.1$), **($p < 0.01$), ***($p < 0.001$), ****($p < 0.0001$). Error bars: mean ± SD ($n = 3$).

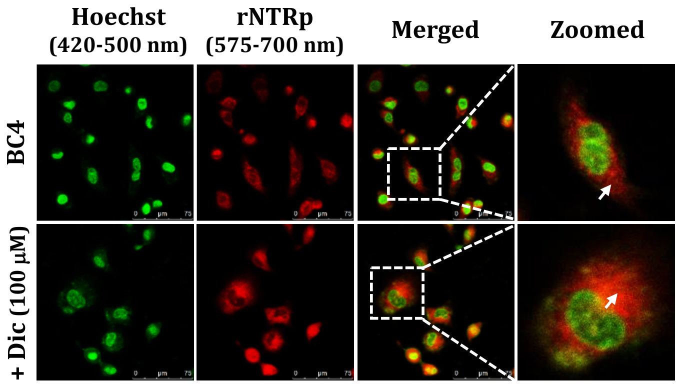

Figure S9. Images of A549 cells incubated with **BC4** in the absence and presence of a nitroreductase inhibitor dicoumarol (Dic). The cells were co-incubated with **BC4** (5.0 µM) and Hoechst (10 µM) in normoxic conditions for 1 h, followed by the addition of Dic. Images were obtained under excitation at 405 nm and emission collection in two channels: Hoechst in green (420–500 nm), **BC4** in red (575–700 nm). Images were obtained 5 min after the addition of Dic.

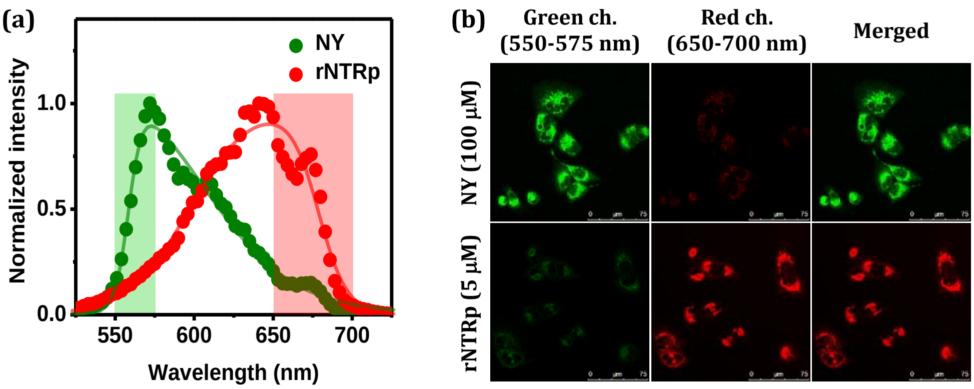

Figure S10. (a) Emission spectra of **NY** and **rNTRp** in A549 cells. (b) CLSM image of A549 cells co-incubated with **NY** (100 µM) and **rNTRp** (5.0 µM), observed in green (550–575 nm) and red (650–700 nm) channels, respectively under 488 nm excitation.

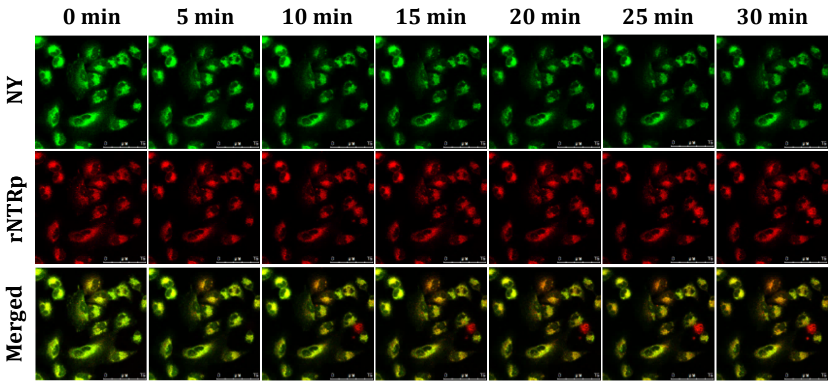

Figure S11. CLSM images of A549 cells that were co-incubated with **NY** (100 µM) and **rNTRp** (5.0 µM) for 6 h, followed by replacing the medium with pH 7.4 PBS containing 10% MeOH. Images were obtained in a five-minute interval up to 30 min.

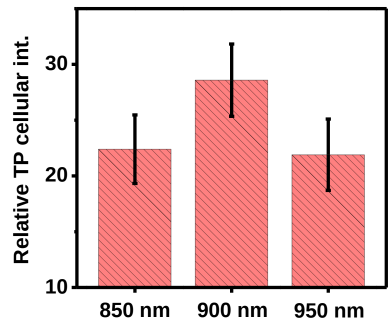

Figure S12. Two-photon cellular intensities of **rNTRp** depending on the excitation wavelengths. A549 cells were incubated with **rNTRp** (5.0 μM) for 1 h and then subjected to imaging under two-photon excitation at different wavelengths and emission collection from 600–650 nm.

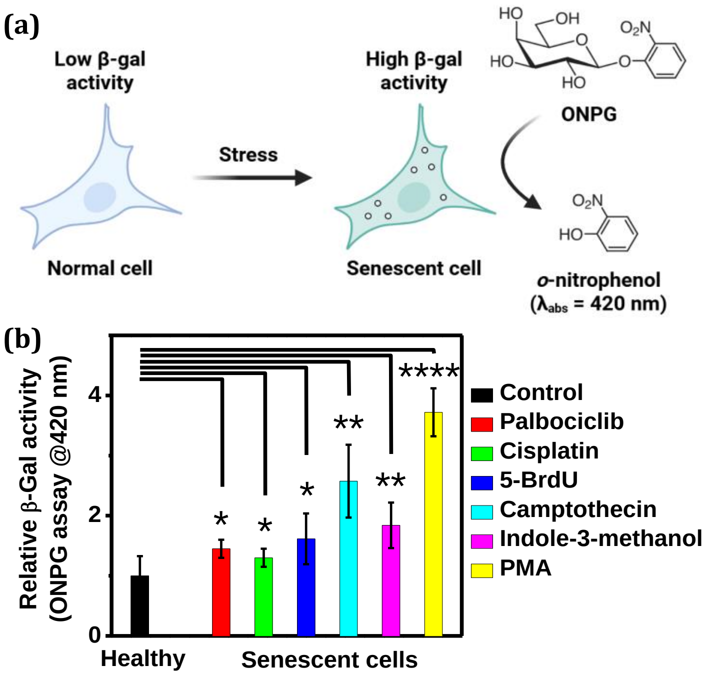

Figure S13. Investigation on the association of the red-NTR activity with elevated ROS levels. A549 cells, seeded on to 96 well plate, were separately treated with palbociclib (10 μM, for 96 h), cisplatin (15 μM, for 72 h), 5-BrdU (100 μM, for 48 h), camptothecin (200 nM, for 48 h), indole-3-methanol (200 μM, for 48 h), and PMA (1.0 μg/mL, for 24 h). After being washed with 10 mM pH 7.4 PBS, the cells were treated with ONPG (1.0 mg/ml, dissolved in Z-buffer pH 6.0) to specifically detect the senescence-associated β-galactosidase activity. After 3 h-incubation at 37 °C, the absorbance from the enzymatic product, *o*-nitrophenol, was measured at 420 nm. The measured absorbance was calibrated with the cell viability to assess the relative β-galactosidase activity of each sample.

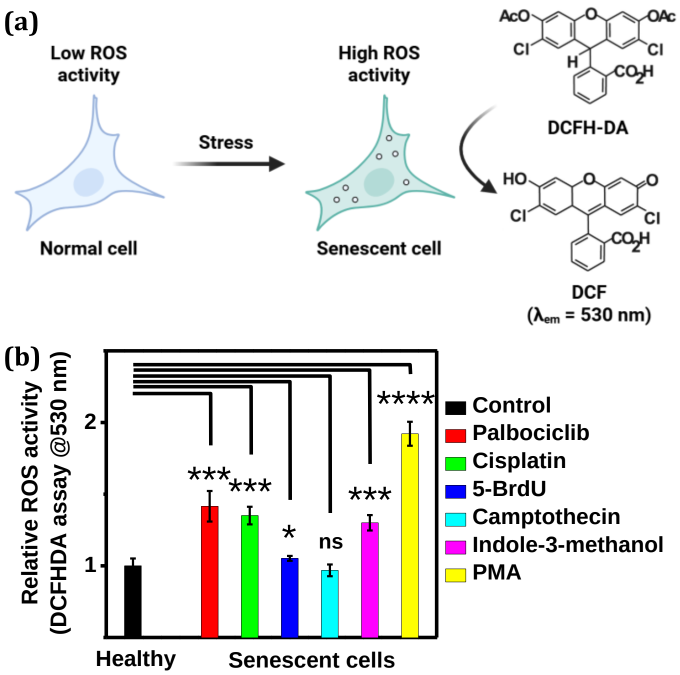

Figure S14. Supporting data for the senescence induction in the different A549 cell line models. A549 cells seeded on to a 96-well plate and treated with palbociclib (10 µM, for 96 h); cisplatin (15 µM, for 72 h); 5-BrdU (100 µM, for 48 h); camptothecin (200 nM, for 48 h); indole-3-methanol (200 µM, for 48 h); PMA (1.0 µg/mL, for 24 h). After being washed with PBS, the cells were treated with DCFH-DA (10.0 µM) for 1 h and then fluorescence intensity at 530 nm was recorded. The intensity was calibrated with the cell viability to assess the relative ROS activity in the samples.

2. Synthesis

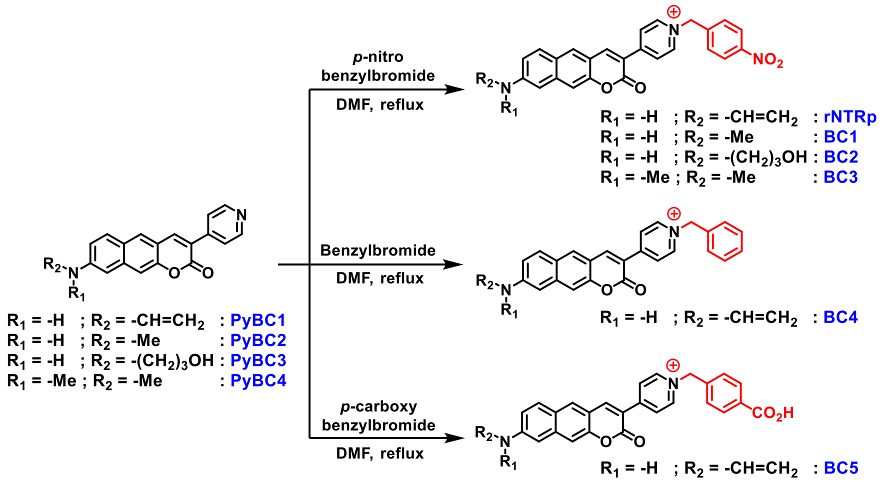

Scheme S2. Synthesis of the compounds used in this study.

PyBC1–4: They were prepared by following the reported procedure (Chem. Sci. **2017**, *8*, 7696).

rNTRp: To a solution of **PyBC1** (33 mg, 0.1 mmol) in DMF (1 mL) was added 4-nitrobenzyl bromide (33 mg, 0.15 mmol, 1.5 equiv.). The solution was heated to 80 °C and stirred for 12 h. DMF was evaporated, and the crude product was washed with ethyl acetate followed by methanol to afford **rNTRp** as a deep brown solid (~75% yield). ^{1}H NMR (DMSO-d_6, 500 MHz, 296 *K*): δ 9.20 (d, *J* = 6.0 Hz, 2H), 8.94 (s, 1H), 8.62 (d, *J* = 6.5 H, 2H), 8.31 (d, *J* = 8.5 Hz, 2H), 8.19 (s, 1H), 7.84 (d, *J* = 9 Hz, 1H), 7.79 (d, *J* = 8.5 Hz, 2H), 7.49 (s, 1H), 7.12–7.08 (m, 2H), 6.78 (s, 1H), 5.98 (s, 2H), 5.98 (m, 1H), 5.32–5.17 (m, 2H), 3.87 (s, 2H). ^{13}C{^{1}H} NMR (DMSO-d_6, 125 MHz, 298 *K*): δ 158.9, 151.4, 150.9, 149.9, 147.9, 147.3, 144.3, 141.3, 138.9, 134.7, 131.4, 130.4, 129.9, 125.7, 124.1, 123.6, 119.1, 116.4, 115.9, 114.2, 108.3, 61.4, 44.7. HRMS (FAB$^+$): m/z, calculated for $C_{28}H_{22}N_3O_4^+$ [M]$^+$ 464.1605; found 464.1613.

BC1–5: These compounds were synthesized by following the above procedure using appropriate **PyBC** derivatives and benzyl bromide derivatives (1.5 equiv.). The crude products were purified by HPLC to afford **BC1–5** as deep-brown solids, which were confirmed by LC-MS (ESI$^+$) analysis: m/z calculated for **BC1** $C_{26}H_{20}N_3O_4^+$ [M]$^+$ 438.1448, found 438.0; m/z calculated for **BC2**, $C_{28}H_{24}N_3O_5^+$ [M]$^+$ 482.1710, found 482.0; m/z calculated for **BC3**, $C_{27}H_{22}N_3O_4^+$ [M]$^+$ 452.1605, found 452.0; m/z calculated for **BC4**, $C_{28}H_{23}N_2O_2^+$ [M]$^+$ 419.1754, found 419.0; m/z calculated for **BC5**, $C_{29}H_{23}N_2O_4^+$ [M]$^+$ 463.1652, found 463.0.

NY: NY was prepared following the reported procedure (*Anal. Chem.* **2022**, *94*, 7272–7277). m/z calculated for $C_{27}H_{27}N_2O_3^+$ [M]$^+$ 427.2016, found 427.0.

3. NMR data

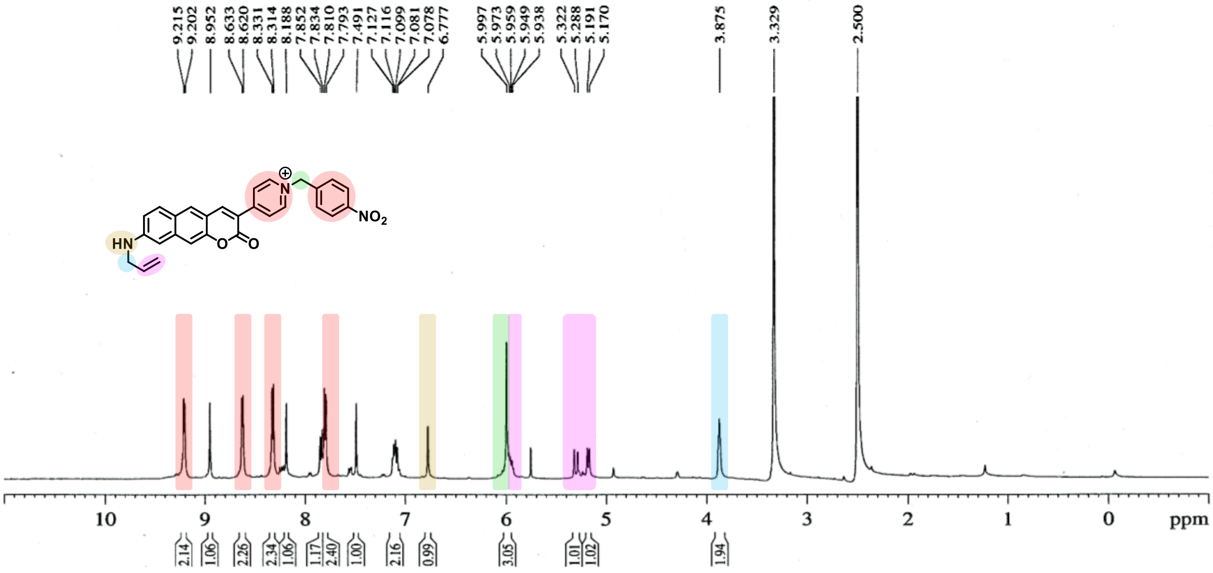

^{1}H NMR of **rNTRp** in DMSO-d_6 (500 MHz, 25 °C)

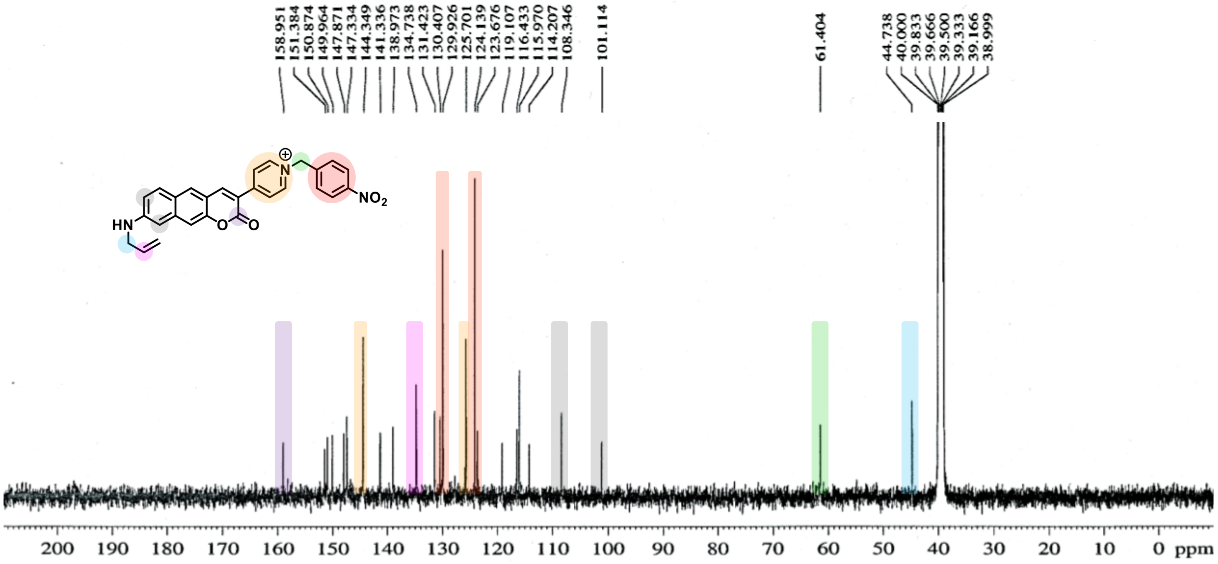

^{13}C{^{1}H} NMR of **rNTRp** in DMSO-d_6 (125 MHz, 25 °C)

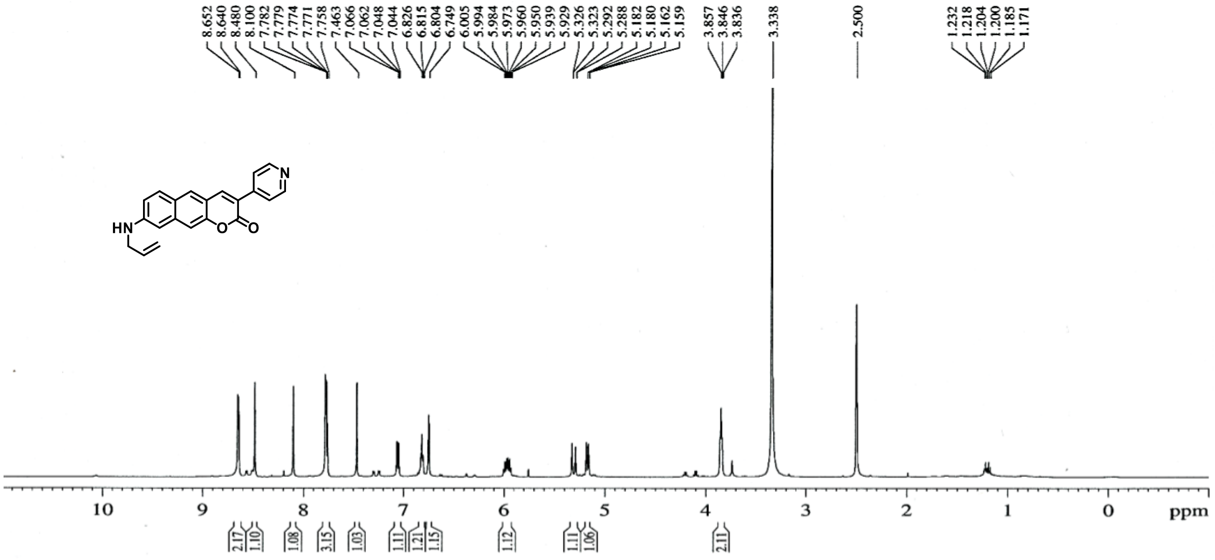

^{1}H NMR of **PyBC1** in DMSO-d_6 (500 MHz, 25 °C)

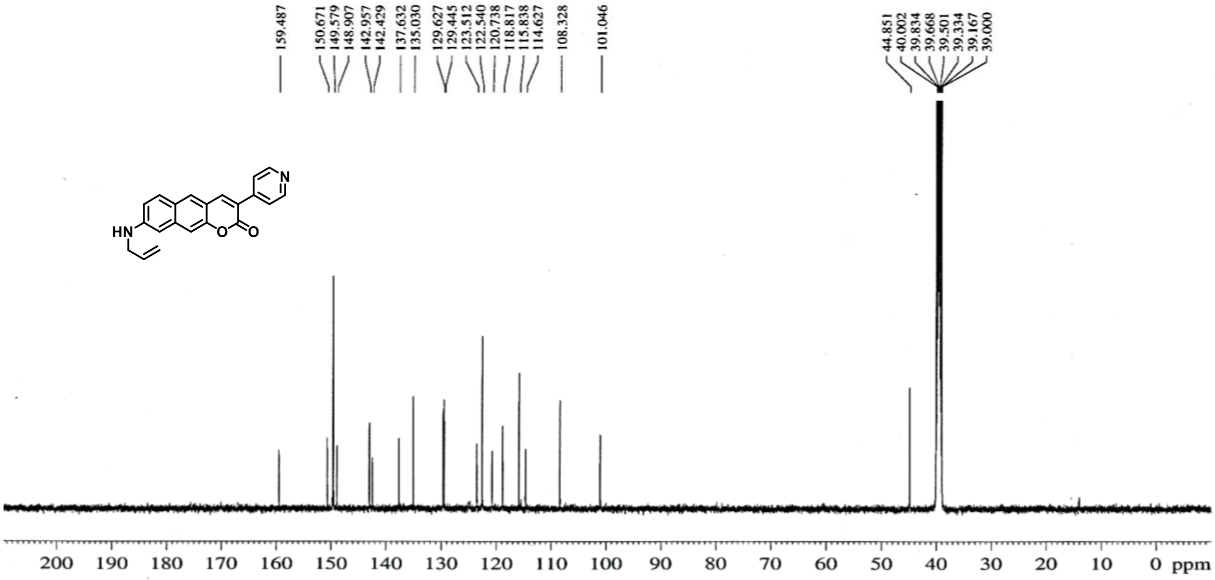

^{13}C{^{1}H} NMR of **PyBC1** in DMSO-d_6 (125 MHz, 25 °C)

4. HRMS data

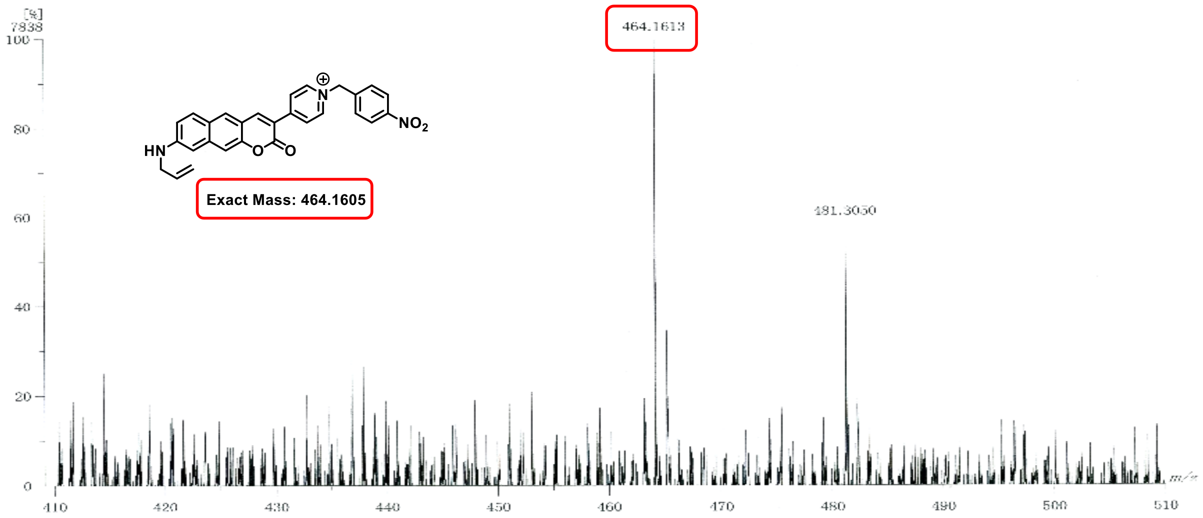

HRMS (FAB+) spectra of **rNTRp**

5. LC-MS characterization

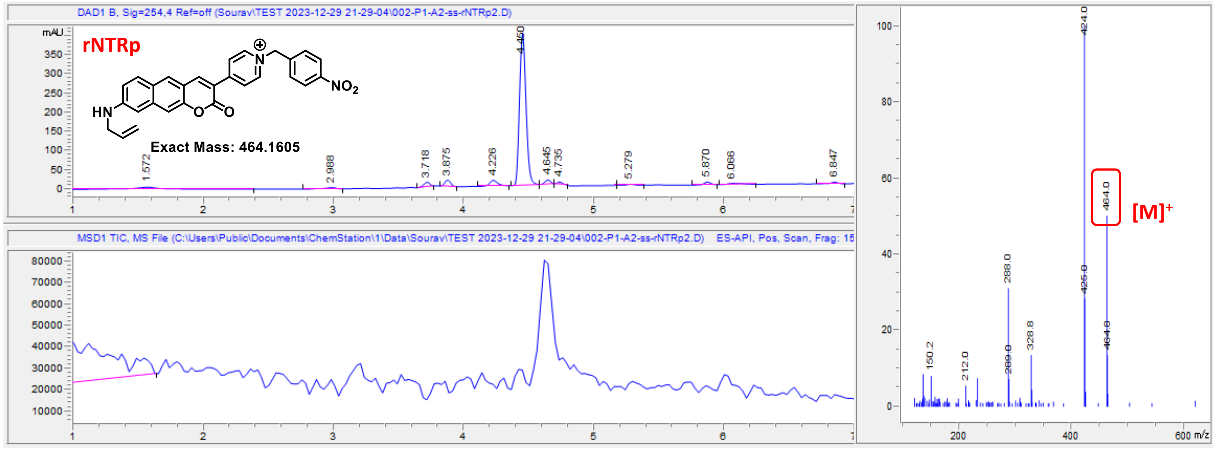

LC-MS (ESI+) spectra of **rNTRp**

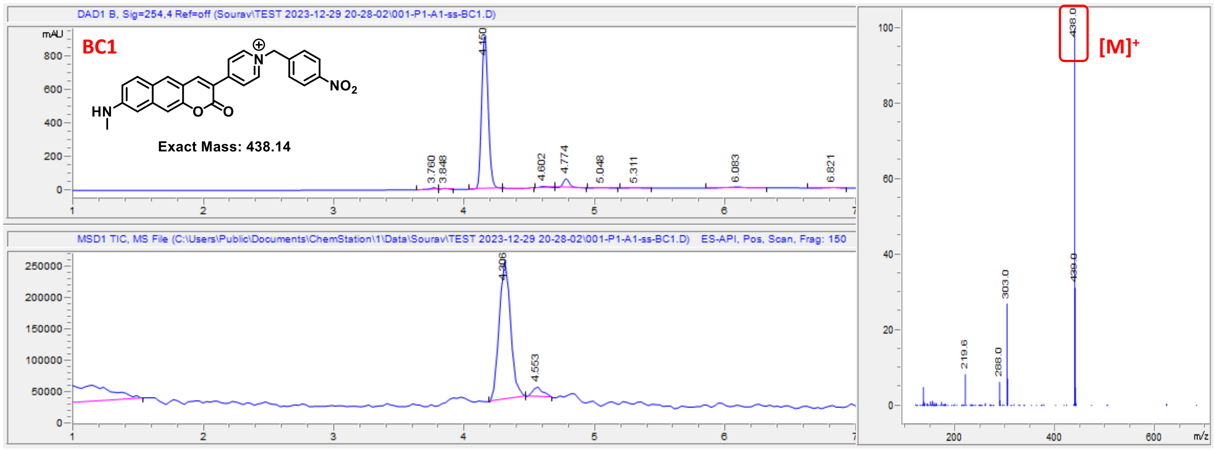

LC-MS (ESI+) spectra of **BC1**

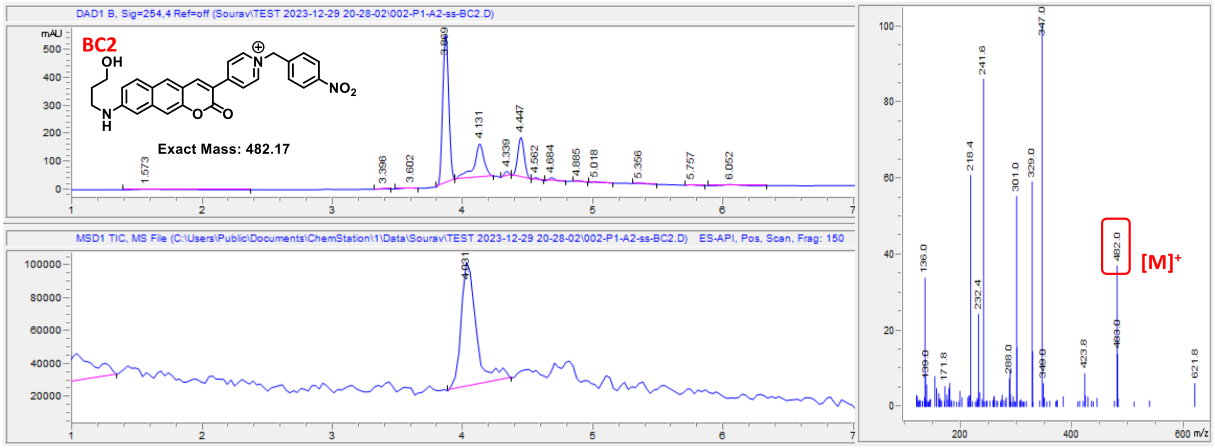

LC-MS (ESI+) spectra of **BC2**

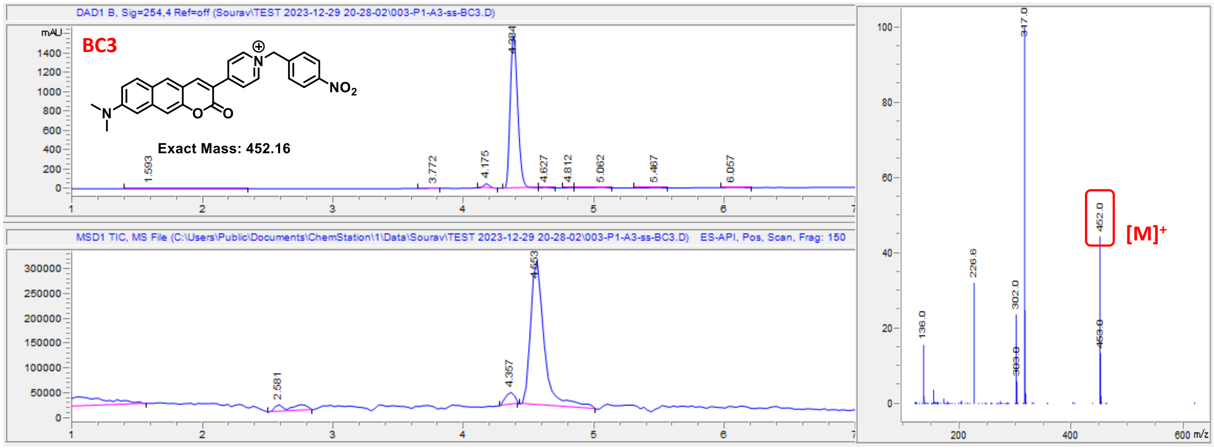

LC-MS (ESI+) spectra of **BC3**

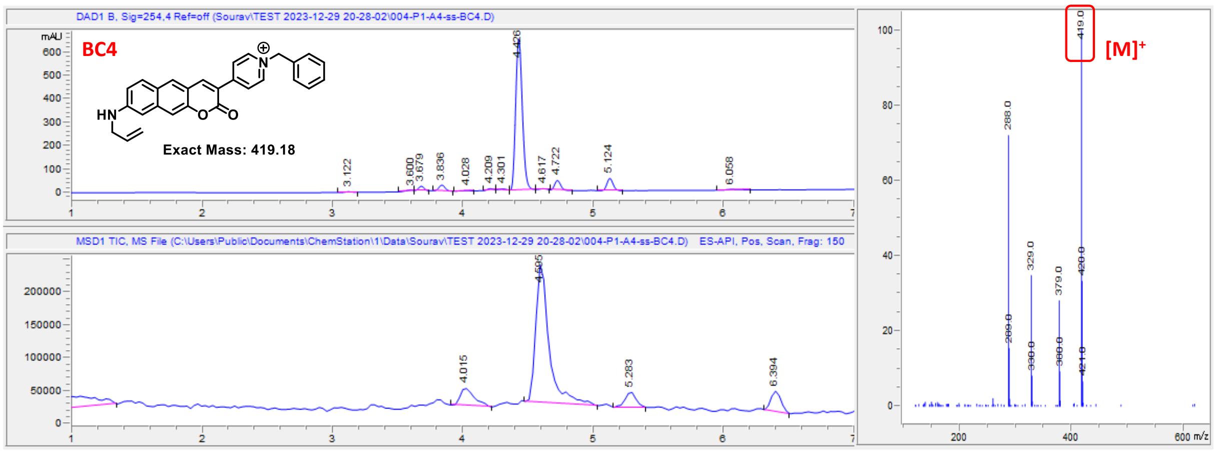

LC-MS (ESI+) spectra of **BC4**

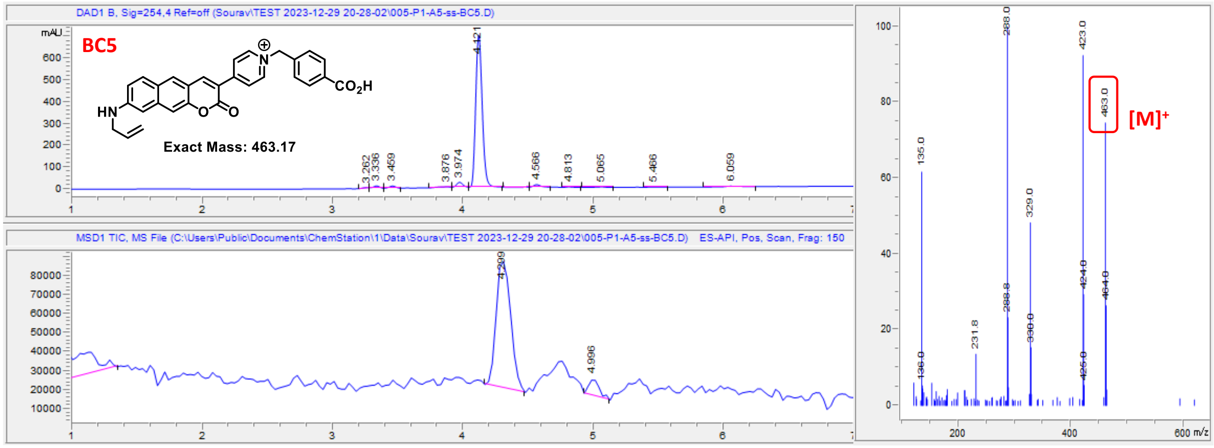

LC-MS (ESI+) spectra of **BC5**

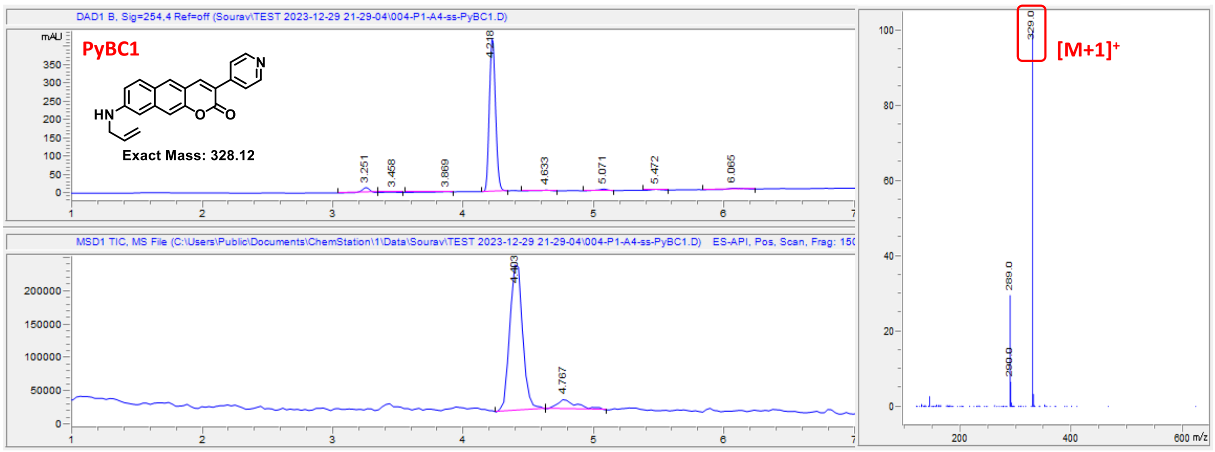

LC-MS (ESI$^+$) spectra of **PyBC1**

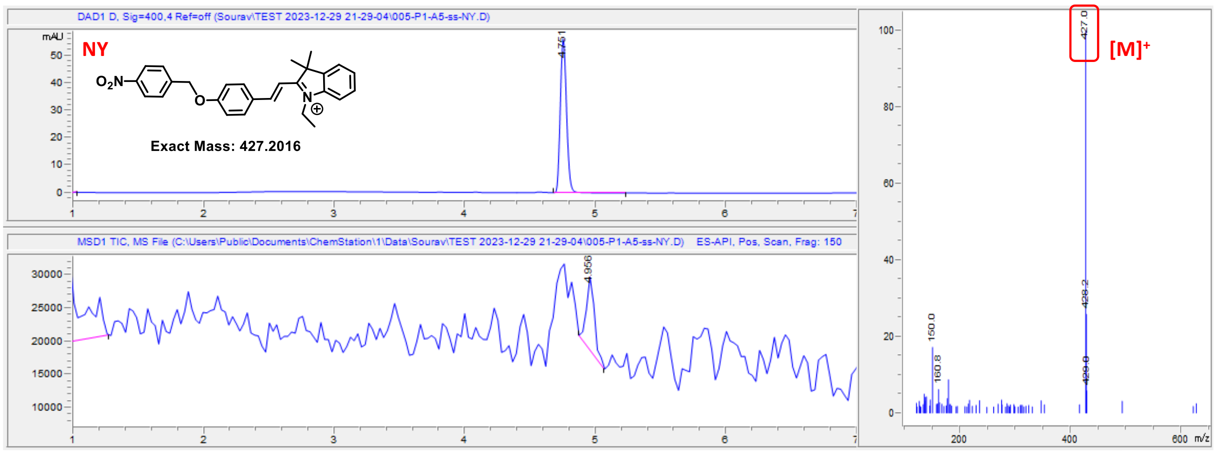

LC-MS (ESI$^+$) spectra of **NY**
